# Functional regeneration and repair of tendons using biomimetic scaffolds loaded with recombinant periostin

**DOI:** 10.1038/s41467-021-21545-1

**Published:** 2021-02-26

**Authors:** Yu Wang, Shanshan Jin, Dan Luo, Danqing He, Chunyan Shi, Lisha Zhu, Bo Guan, Zixin Li, Ting Zhang, Yanheng Zhou, Cun-Yu Wang, Yan Liu

**Affiliations:** 1 Laboratory of Biomimetic Nanomaterials, Department of Orthodontics, Peking University School and Hospital of Stomatology, National Engineering Laboratory for Digital and Material Technology of Stomatology, Beijing Key Laboratory of Digital Stomatology, Beijing, China; 2 State Key Laboratory of Heavy Oil Processing, College of New Energy and Materials, Beijing Key Laboratory of Biogas Upgrading Utilization, China University of Petroleum (Beijing), Beijing, China; 3 Department of Radiology, Beijing Anzhen Hospital, Beijing Institute of Heart, Lung & Vascular Diseases, Capital Medical University, Beijing, China; 4 Beijing National Laboratory for Molecular Science, Institute of Chemistry, Chinese Academy of Sciences, Beijing, China; 5 Laboratory of Molecular Signaling, Division of Oral Biology and Medicine, School of Dentistry and Jonsson Comprehensive Cancer Center, University of California Los Angeles, Los Angeles, CA United States

**Keywords:** Biomedical materials, Ageing, Regeneration, Biomedical engineering, Bioinspired materials

## Abstract

Tendon injuries disrupt the balance between stability and mobility, causing compromised functions and disabilities. The regeneration of mature, functional tendons remains a clinical challenge. Here, we perform transcriptional profiling of tendon developmental processes to show that the extracellular matrix-associated protein periostin (Postn) contributes to the maintenance of tendon stem/progenitor cell (TSPC) functions and promotes tendon regeneration. We show that recombinant periostin (rPOSTN) promotes the proliferation and stemness of TSPCs, and maintains the tenogenic potentials of TSPCs in vitro. We also find that rPOSTN protects TSPCs against functional impairment during long-term passage in vitro. For in vivo tendon formation, we construct a biomimetic parallel-aligned collagen scaffold to facilitate TSPC tenogenesis. Using a rat full-cut Achilles tendon defect model, we demonstrate that scaffolds loaded with rPOSTN promote endogenous TSPC recruitment, tendon regeneration and repair with native-like hierarchically organized collagen fibers. Moreover, newly regenerated tendons show recovery of mechanical properties and locomotion functions.

## Abstract (web summary)

The regeneration of functional tendons remains a clinical challenge. Here the authors develop a biomimetic scaffold loaded with recombinant periostin and demonstrate its functionality in promoting tendon stem/progenitor cell recruitment and tenogenic differentiation, and tendon regeneration in a rat full-cut Achilles tendon defect model.

## Introduction

Tendon is a unique mechanosensitive tissue that transmits forces from muscle to bone, enabled by hierarchical organization of highly aligned collagen extracellular matrix (ECM) deposited by tenocytes and tendon stem/progenitor cells (TSPCs)^1^. This hierarchical ECM niche regulates TSPC differentiation and functions^2^. Tendon injuries, characterized by disorganized ECM fibers, often lead to joint instability and compromised functions; they pose a substantial clinical burden on healthcare systems worldwide^3^. However, there are a few therapeutic interventions for functional tendon regeneration due to limited comprehension of the cells and microenvironment that drive tendon regeneration.

TSPCs possess typical stem cell characteristics, including clonogenicity, self-renewal, and multipotency^2,4,5^. In the neonatal stage, the proliferation and differentiation capacities of TSPCs are sufficient to functionally regenerate injured tendons. However, regenerative capacities are greatly diminished in mature tendons, which exhibit hypocellularity, as well as nutrient and oxygen deficiencies^2,3,6^. Attempts to expand adult stem cells in vitro have included hypoxia, three-dimensional culture, and substrate patterning^7–9^. Growth factors such as transforming growth factor beta (TGF-β), insulin like growth factor 1 (IGF-1), platelet-derived growth factor (PDGF), and growth differentiation factor 5 (GDF5) have also been applied to promote tendon injury healing^10,11^. However, due to a lack of knowledge regarding specific regulatory factors during postnatal tendon development, long-term in vitro culture of TSPCs and functional regeneration of tendons have not been achieved. Discoveries regarding developmental mechanisms are critical for developing strategies for tissue engineering and regeneration^12,13^. To identify molecules with the potential to promote tendon regeneration, we compared gene expression changes associated with rat tendon development using transcriptional profiling. We found that periostin (Postn), a secreted ECM protein involved in the regulation of cell–cell and cell–matrix interactions^14^, was expressed at high levels in early rat tendon development and reduced in mature tendons. Postn exerted vital biological functions, including promotion of TSPC stemness and tenogenic differentiation potentials. To mimic the native tendon microenvironment, we developed a biomimetic scaffold with a parallel-aligned collagen structure, which potently supported TSPC growth and tendon formation. Furthermore, using a rat full-cut Achilles tendon defect model, we achieved structural and functional regeneration of tendons by a combination of biomimetic scaffold and recombinant periostin (rPOSTN).

## Results

### Abundant expression of Postn in neonatal tendons

The basic building blocks of tendons are type-I ECM collagen fibers, which are composed of highly parallel-aligned fibrils deposited by tendon cells. The number of proliferating cells was reported to be greater in the tendon ECM niche from 3-day-old mice than from 6-week-old mice^2^. Compared to the 6–8-week-old rat Achilles tendons (postnatal 6–8 weeks, P 6w) that contained a few proliferating cells, neonatal tendons (postnatal 1 day, P 1d) showed very large numbers of cells residing between slender collagen fibrils (Supplementary Fig. 1a). Immunohistochemistry analysis showed that the expression of the proliferation-related gene *Ki67* and tendon lineage-specific genes *Scleraxis* (*Scx*, the earliest identified marker expressed by tendon progenitors) and *Mohawk* (*Mkx*, a key regulator of postnatal tendon maturation)^2,15^, decreased markedly with tendon maturation (Supplementary Fig. 1b). Although the biological events underlying the modulation of embryonic tendon development have been closely studied^16,17^, their ability to orchestrate postnatal tendon maturation in a manner similar to that of tendon regeneration remains largely unknown. For molecular profiling of tendon maturation, mRNA microarray analyses were performed in total tendon tissues harvested from normal neonatal and 6–8-week-old rat Achilles tendons. Overall, >5000 genes showed significant differences in expression between 6–8-week-old and neonatal tendons. Notably, the number of downregulated genes far surpassed that of upregulated genes, with a twofold change in expression level as the cutoff (*P* < 0.05, Supplementary Fig. 1c). To identify sets of genes that distinguished neonatal and 6–8-week-old tendons, gene ontology (GO) analysis and gene set enrichment analysis (GSEA) were performed. The results of GO analysis showed that the 6–8-week-old tendons lacked expression of genes involved in “cell–cell adhesion”, “cell division”, and “stem cell population maintenance” compared to neonatal tendons (Supplementary Fig. 1d). Further GSEA based on Kyoto Encyclopedia Gene and Genomes (KEGG) database revealed marked reductions in expression of genes in the ribosome, DNA replication, and cell cycle categories (Supplementary Fig. 1e). These findings implied that the downregulated factors during tendon maturation might have vital biological functions. Therefore, this study focused on the downregulated genes to identify specific genes that may modulate TSPC functions, with the aim of finding methods to improve tendon regeneration and repair.

**Fig. 1:**
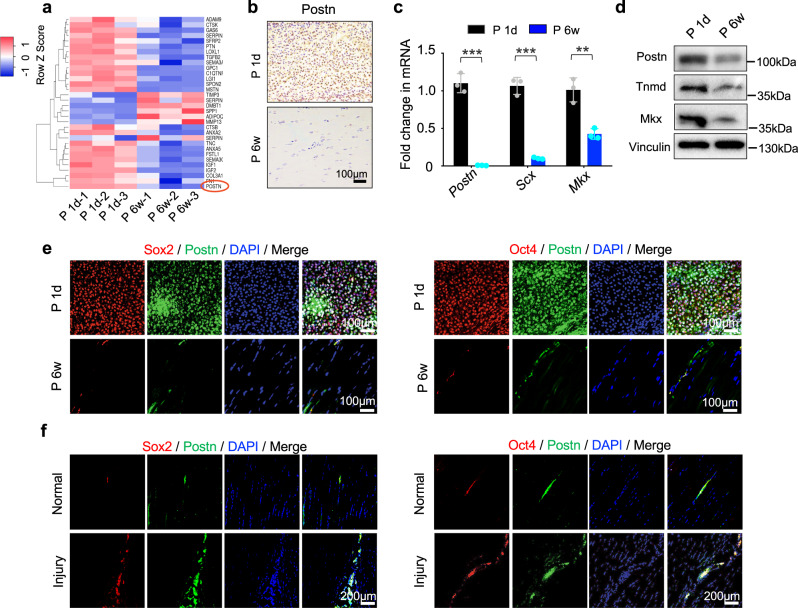
Postn is highly expressed in postnatal tendon development and endogenous tendon injury repair. **a** Heatmap of the 32 differentially expressed gene profiles between neonatal and 6–8-week-old tendons (*n* = 3 rats per group). **b** Immunohistochemistry of Postn expression in the P 1d and P 6w Achilles tendons (*n* = 3 rats per group). **c**, **d** RT-qPCR of *Postn, Scx,* and *Mkx* expression (**c**, *n* = 3 independent experiments, by two-tailed Student’s *t* test: ***P < 0.001, **P < 0.01) and Western blotting of *Postn*, Tnmd, Mkx at P 1d and P 6w (**d**). **e** Immunofluorescence staining of Postn, Sox2, and Oct4 in the rat Achilles tendon between P 1d and P 6w groups (*n* = 5 rats per group). **f** Immunofluorescence staining of Postn, Sox2, and Oct4 in normal and injured Achilles tendons at 1 week postoperatively (*n* = 5 rats per group). Data are presented as mean ± SD. Exact *P* values were given in the Source Data file.

TSPC fate is controlled by a specific ECM niche^2,9,18^. To screen for candidate genes that may confer greater regenerative capacity, “external stimulus”, “matrisome”, and “extracellular space” were intersected; 32 candidate genes were selected, among which *Postn* (the gene encoding Postn) showed the greatest reduction in expression in 6–8-week-old tendons (Fig. 1a; Supplementary Fig. 1f). Microarray analysis identified 88 *Postn*-linked genes that showed differential expression between neonatal and 6–8-week-old tendons (Supplementary Fig. 1g). To confirm these results, reverse transcription quantitative polymerase chain reaction (RT-qPCR), Western blotting, and immunohistochemistry analyses were performed; these studies revealed that the expression levels of *Postn*, *Scx*, and *Mkx* decreased consistently from P 1d to P 6w (Fig. 1b–d). It has been shown that Postn is vital for modulating stemness of stem cells, including cancer stem cells, skeletal stem cells, and neutral stem cells^14,19^. Here, Postn and stemness-related markers Sox2 and Oct4 were found to be highly coexpressed in neonatal tendons; their expression levels were markedly reduced in 6–8-week-old tendons (Fig. 1e). Notably, a recent study indicates that the tendon sheath also harbors TSPCs that contribute significantly to tendon repair^20^; similarly, the present study revealed abundant Postn^+^Sox2^+^ and Postn^+^Oct4^+^ cells in the sheaths of neonatal tendons (Supplementary Fig. 1h). Therefore, we postulated that Postn plays a role in homeostasis and TSPC functions in the ECM-rich environment of the tendon niche.

Endogenous tendon stem cells expressing the stem cell markers CD146 or CD105 are activated in the early stage of tendon injury repair^2,4,5^. Therefore, we compared Postn expression in Achilles tendons under physiological and injured conditions. Immunofluorescence staining showed that the accumulation of Postn-positive cells at sites of injury after 7 days, and a large proportion of these cells coexpressed CD146, Sox2, Oct4, and Ki67. Accumulation of these cells rarely occurred in the physiological state (Fig. 1f, Supplementary Fig. 1i). To further determine whether Postn was associated with tendon cell stemness, primary TSPCs were isolated and identified using flow cytomerty. The isolated TSPCs expressed mesenchymal surface markers, including CD90, CD105, and CD44, but were negative for hematopoietic cell markers CD45 and CD34^2^ (Supplementary Fig. 2a). Toluidine blue staining was performed to confirm chondrogenesis of TSPCs in vitro (Supplementary Fig. 2b). Western blotting analysis indicated that TSPCs from neonatal tendons showed much higher expression levels of Sox2 and Oct4, compared to 6–8-week-old tendons (Supplementary Fig. 2c). Taken together, these findings suggested that the ECM-associated protein Postn might modulate the biological functions of TSPCs.

### Promotion of TSPC stemness, proliferation, and tenogenesis by Postn through the PIK3-AKT axis

**Fig. 2:**
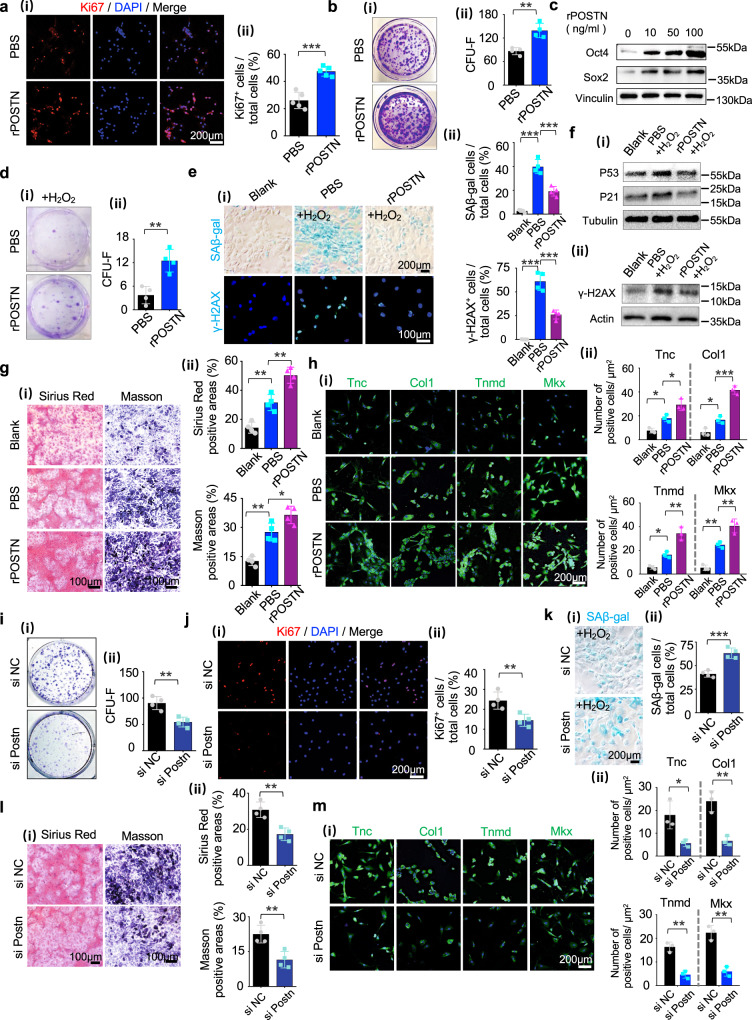
Postn promotes TSPC stemness and tenogenic differentiation potentials in early passage in vitro. **a** Immunofluorescence staining (i) and semi-quantification (ii) of Ki67 in PBS- and rPOSTN-treated TSPCs (*n* = 5 biologically independent samples, by two-tailed Student’s *t* test: ***P < 0.001). **b** (i) CFU-F assay of PBS- and rPOSTN-treated TSPCs. (ii) Semi-quantification of (i) (*n* = 4 biologically independent samples, by two-tailed Student’s *t* test: **P < 0.01). **c** Western blotting of Sox2 and Oct4 protein expression in TSPCs after rPOSTN treatment at different concentrations. **d** (i) CFU-F assay for assessing the anti-oxidative stress capacity of PBS- and rPOSTN-treated TSPCs under exposure to H_2_O_2_. (ii) Semi-quantification of (i) (*n* = 4 biologically independent samples, by two-tailed Student’s *t* test: **P < 0.01). **e** (i) SAβ-gal staining (top panel) and immunofluorescence staining of DNA injury-related protein γ-H2AX (bottom panel) of PBS- and rPOSTN-treated TSPCs suffering from H_2_O_2_ stimulation. Blank: without H_2_O_2._ The blue cells are senescent cells. (ii) Semi-quantification of (i) (*n* = 4 biologically independent samples, by one-way ANOVA with Tukey’s post hoc test: ***P < 0.001). **f** Western blotting of senescence-related protein P53, P21, and γ-H2AX of PBS- and rPOSTN-treated TSPCs suffering from H_2_O_2_ stimulation. **g** (i) Sirius Red staining (left panel) and Masson’s trichrome staining (right panel) of PBS- and rPOSTN-treated TSPCs. The pink and mazarine areas are positively stained respectively. (ii) Semi-quantification of (i) (*n* = 4 biologically independent samples, by one-way ANOVA with Tukey’s post hoc test: **P < 0.01, *P < 0.05). **h** Immunofluorescence staining (i) and semi-quantification (ii) of tenogenic markers Tnc, Col1, Tnmd, and Mkx in PBS- and rPOSTN-treated TSPCs (*n* = 3 biologically independent samples, by one-way ANOVA with Tukey’s post hoc test: ***P < 0.001, **P < 0.01, *P < 0.05). **i** (i) CFU-F assay of si NC- and si Postn-treated TSPCs. Si NC: negative control siRNA. (ii) Semi-quantification of (i) (*n* = 4 biologically independent samples, by two-tailed Student’s *t* test: **P < 0.01). **j** (i) Immunofluorescence staining of Ki67 of si NC- and si Postn-treated TSPCs. (ii) Semi-quantification of (i) (*n* = 4 biologically independent samples, by two-tailed Student’s *t* test: **P < 0.01). **k** (i) Representative images of SAβ-gal staining of si NC- and si Postn-treated TSPCs suffering from H_2_O_2_ stimulation. The blue cells are senescence cells. (ii) Semi-quantification of (i) (*n* = 4 biologically independent samples, by two-tailed Student’s *t* test: ***P < 0.001). **l** (i) Sirius Red staining (left panel) and Masson’s trichrome staining (right panel) of si NC- and si Postn-treated TSPCs (*n* = 4 biologically independent samples, by two-tailed Student’s *t* test: **P < 0.01). **m** (i) Immunofluorescence staining of tenogenic markers Tnc, Col1, Tnmd, and Mkx in si NC- and si Postn-treated TSPCs. (ii) Semi-quantification of (i) (*n* = 3 biologically independent samples, by two-tailed Student’s *t* test: **P < 0.01, *P < 0.05). Data are represented as mean ± SD. Exact *P* values were given in the Source Data file.

TSPCs have been shown to exhibit stem cell functions in vitro, including self-renewal, clonogenicity, and multipotency^2,4,5^. To investigate whether Postn influences the phenotypic characteristics of TSPCs, rPOSTN was added to the culture medium. After 3 days, rPOSTN-treated TSPCs showed markedly elevated proliferation capacity, compared with the untreated group, as indicated by the greater number of Ki67^+^ cells (Fig. 2a). Colony formation assay showed that the rPOSTN-treated TSPCs had a higher self-renewal capacity than the untreated TSPCs, evidenced by the increases in number and diameter of the colony-forming unit fibroblasts (CFU-Fs) (Fig. 2b). Moreover, rPOSTN promoted the expression of Sox2 and Oct4 in TSPCs (Fig. 2c), suggesting that rPOSTN may aid in maintenance of TSPC stemness. The excessive oxidative stress caused by reactive oxygen species is presumed to impair healing or regeneration of injured tissue by triggering deleterious processes, such as necrosis, inflammation, and fibrotic scarring^21,22^. To resemble this condition in vitro, we treated TSPCs with 200 μM hydrogen peroxide (H_2_O_2_), which markedly impaired their colony formation capacity. However, rPOSTN could significantly attenuate the inhibition of CFU-F formation by H_2_O_2_ (Fig. 2d). Notably, senescence-associated beta-galactosidase (SAβ-gal) staining revealed that rPOSTN could inhibit TSPC replicative senescence. DNA damage associated γ-H2AX and p53/p21 protein levels were markedly suppressed under safeguarding interference by rPOSTN (Fig. 2e, f). These findings indicated that rPOSTN protected TSPCs against premature senescence and DNA damage induced by H_2_O_2_ exposure^23,24^. In addition, the multilineage differentiation capacity of TSPCs pretreated with rPOSTN was evaluated in different induction media without rPOSTN. rPOSTN-treated TSPCs exhibited enhanced tenogenic differentiation in tenogenic-inducing media without rPOSTN, as revealed by staining with Sirius Red and Masson’s trichrome (Fig. 2g). Immunofluorescence analysis also confirmed that the expression levels of tenogenic markers, including Tnc, Col1, Tnmd, and Mkx, were significantly increased in TSPCs pre-treated with rPOSTN (Fig. 2h). Moreover, TSPCs pre-treated with rPOSTN had enhanced osteogenic and adipogenic differentiation of TSPCs (Supplementary Fig. 2d, e). To determine whether Postn knockdown also affected the phenotypic characteristics of TSPCs, its expression was silenced with small interfering RNA (siRNA) against Postn (si Postn). si Postn potently reduced the expression of Postn, as determined by Western blotting (Supplementary Fig. 2f). Postn knockdown reduced expression levels of Oct4 and Sox2 (Supplementary Fig. 2f); it also partially impaired the self-renewal capacity, proliferation capacity, oxidative stress resistance capacity, and multi-lineage differentiation potentials of TSPCs (Fig. 2i–m, Supplementary Fig. 2g–i).

**Fig. 3:**
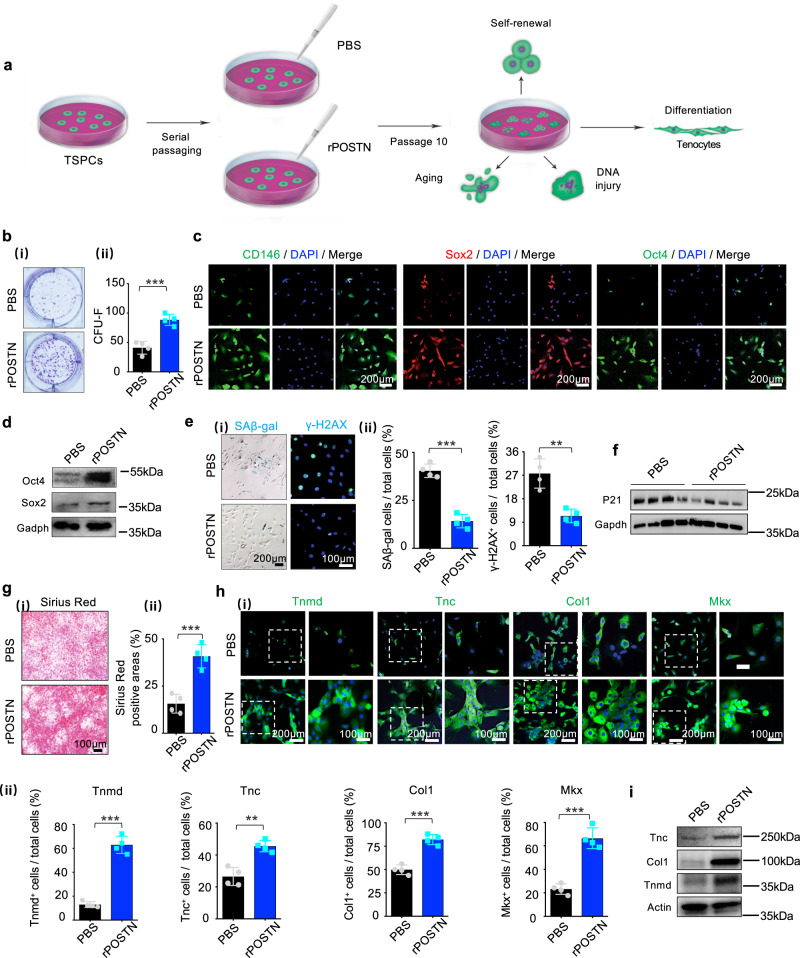
Postn maintains TSPC phenotype and functions after long-term passage in vitro. **a** Schematic of the TSPC serial passaging. **b** (i) CFU-F assay of PBS- and rPOSTN-treated TSPCs. (ii) Semi-quantification of (i) (*n* = 4 biologically independent samples). **c** Immunofluorescence staining of stemness-related markers Sox2 and Oct4, and tendon stem cell marker CD146 of PBS- and rPOSTN-treated TSPCs (*n* = 4 biologically independent samples). **d** Western blotting of Sox2 and Oct4 protein levels of PBS- and rPOSTN-treated TSPCs. **e** (i) SAβ-gal staining (left panel) and immunofluorescence staining of DNA injury-related protein γ-H2AX (right panel) of PBS- and rPOSTN-treated TSPCs. The blue cells are senescent cells. (ii) Semi-quantification of (i) (*n* = 4 biologically independent samples). **f** Western blotting of P21 protein levels of PBS- and rPOSTN-treated TSPCs. **g** (i) Sirius Red staining of PBS- and rPOSTN-treated TSPCs in the tenogenic medium after 14 days. (ii) Semi-quantification of (i) (*n* = 4 biologically independent samples). **h** (i) Immunofluorescence staining of tenogenic markers Tnc, Col1, Tnmd, and Mkx in PBS- and rPOSTN*-*treated TSPCs. (ii) Semi-quantification of (i) (*n* = 4 biologically independent samples). **i** Western blotting of Tnc, Col1, and Tnmd protein levels of PBS- and rPOSTN-treated TSPCs in tenogenic medium. Data are represented as mean ± SD. Exact *P* values were calculated by two-tailed Student’s *t* test and given in the Source Data file. ***P < 0.001, **P < 0.01.

The greatest obstacle to the application of adult stem cells in tissue engineering is that they undergo spontaneous differentiation and lose their regenerative potential after serial passages in vitro^25,26^. To investigate whether rPOSTN administration could ameliorate this TSPC hypofunction, 10 serial passages of TSPCs isolated from 6–8-week-old rats were performed; rPOSTN was found to aid in maintenance of CFU-F formation (Fig. 3a, b). rPOSTN-treated TSPCs retained high expression levels of CD146, Sox2, and Oct4, whereas untreated TSPCs gradually lost expression of these genes, as confirmed by immunofluorescence and western blotting analyses (Fig. 3c, d). Previous studies showed that cellular replicative senescence in long-term culture in vitro may pose considerable problems with regard to stem cell hypofunction^25,26^. SAβ-gal staining revealed that rPOSTN could inhibit TSPC replicative senescence up to the 10th serial passage (Fig. 3e). Consistently, treatment with rPOSTN markedly suppressed γ-H2AX and p21 expression (Fig. 3e, f). The reduced DNA damage and cellular aging of TSPCs by rPOSTN intervention may be positively correlated with their tenogenic differentiation potentials^18,27^. Long-term passage led to weak Sirius Red staining, as well as marked reductions in the expression levels of tendon-specific genes (e.g., Tnmd, Tnc, Col1, and Mkx); these effects were ameliorated by rPOSTN (Fig. 3g–i).

**Fig. 4:**
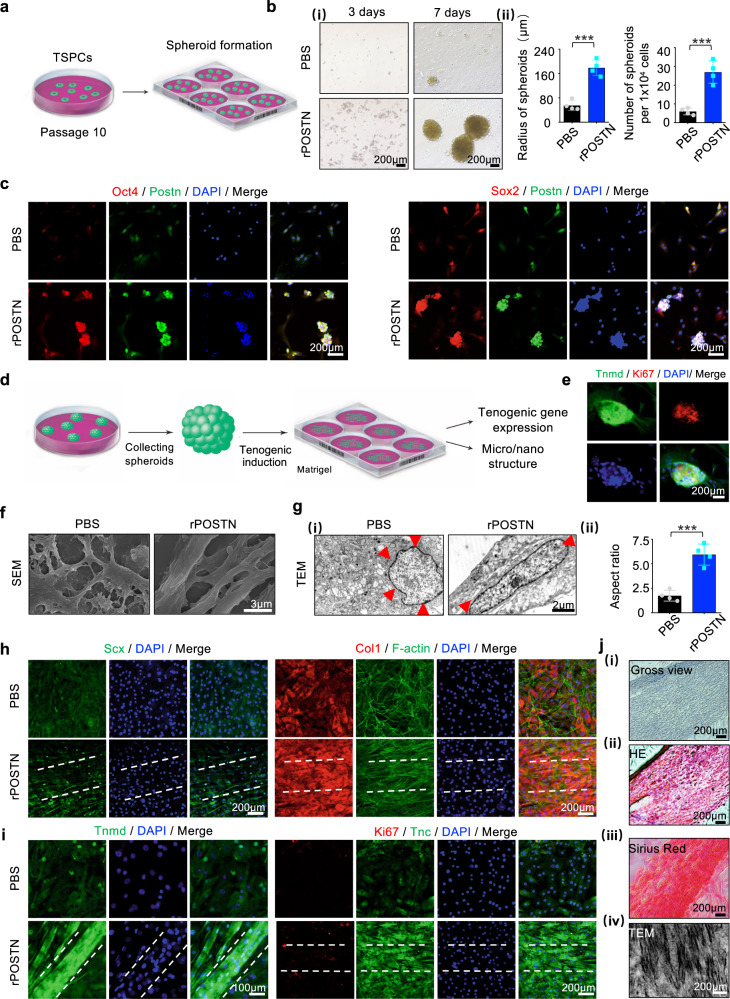
Postn promotes TSPC spheroid formation and maintains tenogenic potentials during long-term passage. **a** Schematic of three-dimensional spheroid formation of TSPCs at the 10th passage. **b** (i) Microscopic images of three-dimensional spheroids of PBS- and rPOSTN-treated TSPCs at 3 days (left panel) and 7 days (right panel). (ii) Semi-quantification of (i) (*n* = 4 biologically independent samples). **c** Immunofluorescence staining of Sox2, Oct4, and Postn of PBS- and rPOSTN-treated TSPCs. **d** Schematic of the differentiation of three-dimensional spheroids. **e** Immunofluorescence staining of Tnmd and Ki67 of spheroids in the tenogenic medium at 3 days. **f** SEM microstructure of collagen fibrils of PBS- and rPOSTN-treated TSPCs. **g** (i) TEM nucleus of PBS- and rPOSTN-treated TSPCs. (ii) Semi-quantification of (i) (*n* = 4 biologically independent samples). **h**, **i** Immunofluorescence staining of Scx, Col1, and F-actin of PBS- and rPOSTN-treated TSPCs on day 14, and Tnmd, Ki67, and Tnc on day 21. **j** (i) Microscopic images, (ii) HE staining, (iii) Sirius Red staining and (iv) TEM of parallel-aligned collagen structure in vitro at 21 days. Data are represented as mean ± SD. Exact *P* values were calculated by two-tailed Student’s *t* test and given in the Source Data file. ***P < 0.001.

The spheroid-forming capacity of stem cells could indicate their own stemness dynamics, and stem cells with a spheroid architecture usually exhibit an excellent self-renewal capacity^28,29^. Three-dimensional cellular spheroids or organoids have been widely utilized to mimic tissue constructs, promote cell differentiation, and serve as disease screening models^30,31^. To construct multicellular spheroids of TSPCs, standard culture dishes were replaced with low-adhesion culture dishes at the 10th passage (Fig. 4a). Long-term passage led to poor spheroid-forming capacity of TSPCs with (~6 total), whereas rPOSTN-restored TSPCs could assemble into more spheroids (~27 total) with a larger radius (178.78 ± 25.55 µm) (Fig. 4b). Immunofluorescence analysis demonstrated that spheroids formed by rPOSTN-restored TSPCs exhibited high expression levels of Postn colocalized with Sox2 and Oct4 (Fig. 4c). Next, the tenogenic differentiation capacity of these spheroids was evaluated (Fig. 4d). After 3 days of induction, the spheroids showed high expression levels of the tendon-maturation marker Tnmd and extended pseudopodia; however, they did not exhibit typical tendon-like parallel-aligned collagen structures (Fig. 4e). Therefore, spheroids were transferred onto normal-adhesion culture dishes coated with Matrigel supplemented with the tenogenic induction medium. Scanning electron microscopy (SEM) and transmission electron microscopy (TEM) revealed that spheroids formed by the rPOSTN-restored TSPCs could stretch out more longitudinally aligned collagen fibers and cells residing among fibers; these cells also possessed a long and elliptical nucleus with an aspect ratio (AR) of 5.94 ± 1.08. In contrast, untreated TSPCs had a round nucleus; they exhibited intersection without fiber formation (Fig. 4f, g). The high AR of cells is closely related to tenogenesis^32^. Immunofluorescence analysis confirmed that rPOSTN-restored TSPCs expressed the tendon-specific nuclear transcriptional factor Scx and the secreted ECM protein Col1 at high levels after 14 days of induction (Fig. 4h). On day 21, the rPOSTN-restored TSPCs formed parallel-aligned structures with high levels of Ki67, Tnmd, and Tnc; they typically had long and elliptical nuclei (Fig. 4i). Hematoxylin & eosin (HE) and Sirius Red staining, as well as TEM analysis, confirmed that rPOSTN-restored TSPCs gradually formed parallel-aligned collagen fibers (Fig. 4j). Taken together, these findings indicated that spheroids formed by rPOSTN-restored TSPCs retained excellent stem cell capacities of self-renewal, proliferation, and tenogenic differentiation potentials.

Existing studies have shown that Postn induced the activation of phos-phatidylinositol-3-OH kinase (PI3K)-AKT signaling pathway^33,34^. PDGFα regulate the self-renewal of skin adipocyte stem cell through the PIK3-AKT signaling axis^35^. Moreover, it has been reported that AKT could interact with Sox2 or Oct4 to promote the self-renewal of cancer stem-like cells^36^. AKT was found to phosphorylate the pluripotency factor Oct4, and increase its stability, and promote its nuclear localization and its interaction with Sox2, which further boosted the transcription of the core stemness genes Pou5f1 and Nanog^36–38^. Therefore, we postulated that Postn regulated the stemness of TSPCs through the PIK3/AKT axis. First, we found that treatment with rPOSTN remarkably induced the phosphorylation of AKT proteins in TSPCs as revealed by immunofluorescence and Western blotting (Supplementary Fig. 3a, b). In early passage, intervention with LY294002, a specific PIK3/AKT inhibitor, evidently reduced the TSPC colony number (Supplementary Fig. 3c). Moreover, Sirius Red staining showed treatment with LY294002 inhibited TSPC tenogenic differentiation potentials, which was accompanied by reducing protein expression of the tenogenic differentiation marker Tnmd. (Supplementary Fig. 3d, e). Furthermore, during the serial passaging to 6th passage, LY294002 treatment reduced expression levels of Sox2 and Oct4 in TSPCs, as revealed by immunofluorescence staining (Supplementary Fig. 3f, g). Colony formation assay showed LY294002-treated TSPCs exhibited impaired colony-formation capacity (Supplementary Fig. 3h). After short serial passaging, treatment with LY29402 led to weaker Sirius Red staining, as well as distinctly reduced protein expression of Tnmd (Supplementary Fig. 3i, j).

### Facilitation of TSPC tenogenesis by biomimetic parallel-aligned collagen fibrils

**Fig. 5:**
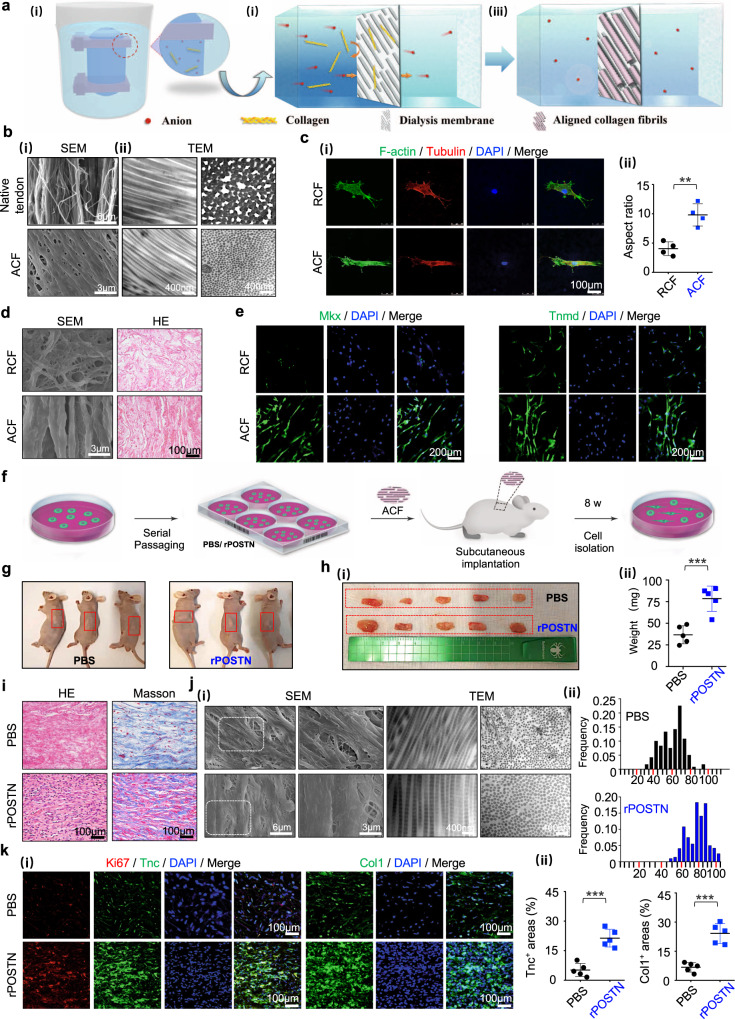
Facilitation of TSPC tenogenesis by biomimetic parallel-aligned collagen fibrils. **a** Schematic of fabrication of parallel-aligned collagen fibers (ACF). **b** (i) SEM of 6–8-week native tendons and ACF. (ii) Corresponding TEM of (i) with longitudinal and cross sections. **c** (i) Immunofluorescence staining of F-actin and Tubulin of the single TSPC on randomly-aligned collagen fibers (RCF) and ACF after 6 h of culture. (ii) Semi-quantification of (i) (*n* = 4 biologically independent samples). **d** (i) SEM and (ii) HE staining of TSPCs on RCF and ACF at 14 days. **e** Immunofluorescence staining of Mkx and Tnmd of TSPCs on RCF and ACF on day 14. **f** Overview of the transplantation protocol of ACF into athymic mice. **g** Representative gross morphology of subcutaneous tissue formed by implantation of ACF with PBS- and rPOSTN-treated TSPCs in the 10th generation after 8 weeks. **h** Macroscopic view (i) and weight (ii) of newly formed tendon-like tissues (*n* = 5 biologically independent samples). **i** HE and Masson’s trichrome staining of sections of neotissues. **j** (i) SEM and TEM of collagen pattern of neotissues. (ii) Distribution of collagen fibril diameters of (i) (*n* = 5 biologically independent samples). **k** (i) Immunofluorescence staining of Ki67, Tnc, and Col1 of neotissues. (ii) Semi-quantification of (i) (*n* = 5 biologically independent samples). Data are represented as mean ± SD. Exact *P* values were calculated by two-tailed Student’s *t* test and given in the Source Data file. ***P < 0.001, **P < 0.01.

The structural anisotropy and hierarchical organization of ECM determine the unique mechanical functions of tendons. To recapitulate the structure–function of tendons, we developed a dynamic diffusion template self-assembly strategy involving two steps: (i) dynamic diffusion to the dialysis membrane surface, and (ii) oriented and ordered self-assembly of collagen molecules on the dialysis membrane template (Fig. 5a). Initially, the salt ions and collagen molecules in the dialysis bag diffused toward external low-concentration solution under the influence of osmotic pressure. The anions and cations penetrated through the porous membrane of the dialysis bag (Mw = 3.5 kDa), while collagen molecules with higher molecular weight (Mw = 115–235 kDa) were trapped on the inner surface of the dialysis bag^39,40^. The dialysis bag with the ordered and regularly arranged fibrous structure (Supplementary Fig. 4a) could be used as a template; it regulated the conformation of the collagen molecules from a disordered manner to a parallel arrangement via Coulomb interactions, achieving the oriented and ordered assembly of collagen molecules^41^. Both dynamic diffusion and dialysis bag template play decisive roles in determining the final superstructure of collagen fibrils. Collagen molecules readily undergo self-assembly into fibrils in a stationary solution environment driven by intermolecular hydrophobic and electrostatic forces^42^, which are much stronger than the attractive forces between collagen and the dialysis bag, and thereby suppressing the induction of the template. In this study, a static assembly method was adopted by simply dropping the collagen solution onto the surface of the dialysis bag. The droplet quickly became turbid because the collagen molecules were trapped in several domains within the solution to form micrometer-scale fibers, which were finally randomly deposited on the substrate surface (RCF, Supplementary Fig. 4a). To overcome the limitations of the super-attractive force between collagen molecules, a dynamically diffusing flow was created under the osmotic pressure. The flow promoted molecular rotation in the velocity gradient plane, while suppressing the self-assembly of collagen^43^. Moreover, collagen molecules were quickly “pushed” to the surface of the dialysis bag; their conformation was adjusted to match the template with the action of fluid disturbance. Finally, the orderly assembly of parallel-aligned collagen fibrils (ACF) was achieved, as revealed by SEM and TEM (Fig. 5b). Moreover, we examined the mechanical properties and controlled release capacity of ACF in vitro. As shown in Supplementary Table 1, ACF possessed the failure force of 33.31 ± 2.06 N, failure stress of 10.60 ± 0.65 N/mm^2^ and elastic modulus of 11.15 ± 1.08 N/mm^2^, which might provide a suitable mechanical support for tendon injuries^44^. A controlled release curve of rPOSTN showed that ACF initiated a burst release within the first 3–7 days, followed by a sustained release profile over a period of 30 days (Supplementary Fig. 4b).

Normal tendons have a highly aligned ECM architecture that becomes disorganized by injury or disease^45^. A number of studies have demonstrated that the topographical geometries and arrangement of ECM could change cellular responses, including cell shape, differentiation, and functions^46–48^. To determine whether geometric characteristics could provide a favorable environment for TSPCs, TSPCs were seeded on the assembled construct for in vitro biological assessment. Immunofluorescence analysis showed that TSPCs on the aligned collagen scaffolds exhibited an elongated shape with a higher AR of 9.83 ± 1.91, similar to native tendon cells. By contrast, disorganized collagen scaffolds yielded polygonal TSPCs with a lower AR of 4.05 ± 1.17 after 12 h in culture (Fig. 5c). SEM and HE staining revealed abundant secreted ECM that was distributed longitudinally on the ACF, whereas RCF led to less random ECM formation (Fig. 5d). Mkx and Tnmd were also regulated by the physical environment. Immunofluorescence analysis showed that the ACF significantly enhanced expression of Mkx and Tnmd, compared to RCF (Fig. 5e). Moreover, expression of the tendon maturation marker Col1 was also upregulated, accompanied by elongated nuclei and cytoskeleton of TSPCs (Supplementary Fig. 4c).

To determine the advantages of parallel structure in the induction of tenogenesis, ACF seeded with TSPCs at 10th passage was implanted into the dorsum of athymic mice; the transplant grafts were harvested after 8 weeks (Fig. 5f). Grossly, the rPOSTN-restored grafts were larger in size and heavier in weight (Fig. 5g, h). HE and Masson’s trichrome staining showed that the rPOSTN-restored grafts yielded collagen fibers with greater density and more cells (Fig. 5i, Supplementary Fig. 4e). Micro/nanostructural examination by SEM and TEM revealed that the rPOSTN-restored group produced longer and thicker collagen fibrils with both an increased average fibril diameter and a larger distribution of diameters (Fig. 5j). Immunofluorescence and immunohistochemistry staining showed that Tnmd, Col1, Scx, and Tnc were expressed at higher levels in the rPOSTN-restored group, compared to the phosphate-buffered saline (PBS) control group (Fig. 5k, Supplementary Fig. 4f, g). Notably, Ki67^+^ cells were abundant in the rPOSTN-restored group, indicating that communication between transplanted rPOSTN-restored TSPCs and the host provided a more suitable environment for proliferation in vivo (Fig. 5k). In addition, cells from the transplanted grafts were isolated at 4 weeks after transplantation; a greater total cell number and more Ki67^+^ cells were present in the rPOSTN-restored group than in the PBS group (Supplementary Fig. 4h, i). Hence, the rPOSTN-restored TSPCs exhibited greater long-term adaptability to their surroundings in vivo for a long time, and ACF could provide a biocompatible survival habitat for transplanted cells.

### Functional regeneration of tendons using ACF loaded with rPOSTN (ACF-rP)

**Fig. 6:**
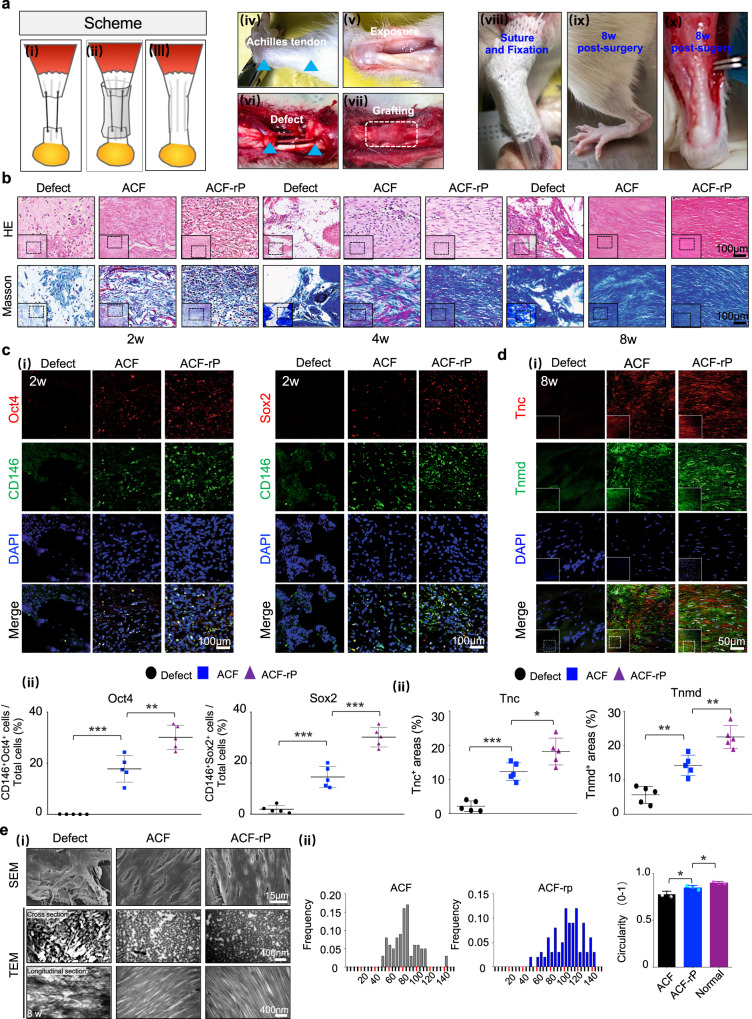
Macro-, micro- and nano- structures of neotendons regenerated by ACF loaded with rPOSTN. **a** (i-iii) Schematic of surgical procedure: (i) A full defect model; (ii) Grafting; (iii) Healing. (iv-x) Gross morphology of an Achilles tendon pre-surgery, surgery, and post-surgery. **b** HE and Masson’s trichrome staining of neotendons from different groups at 2, 4, and 8 weeks postoperatively (*n* = 5 rats per group). **c** (i) Immunofluorescence staining of CD146, Sox2, and Oct4 of neotendons of each group at 2 weeks postoperatively. (ii) Semi-quantification of (i) (*n* = 5 rats per group, by one-way ANOVA with Tukey’s post hoc test: ***P < 0.001, **P < 0.01). **d** (i) Immunofluorescence staining of Tnc and Tnmd of sections of each group at 8 weeks postoperatively. (ii) Semi-quantification of (i) (*n* = 5 rats per group, by one-way ANOVA with Tukey’s post hoc test: ***P < 0.001, **P < 0.01, *P < 0.05). **e** (i) SEM and TEM (transverse and longitudinal) of newly-formed tendon collagen fibrils of each group at 8 weeks postoperatively. (ii) Collagen fibril diameter distribution and circularity in the ACF and ACF-rP groups (*n* = 3 rats per group, by two-tailed Student’s *t* test: *P < 0.05). Data are represented as mean ± SD. Exact *P* values were given in the Source Data file.

For successful tissue engineered therapies, a hierarchical anisotropic scaffold with parallel-aligned collagen fibrils is needed as a mechanical support along with a specific factor guiding tendon injury repair^49^. Here, we established a rat full-cut Achilles tendon defect with 4-mm gap to examine whether Postn could boost a regenerative healing process in combination of ACF (Fig. 6a). At 1, 4, and 8 weeks postoperatively, the ACF and ACF-rP groups maintained contours of tendons, while the Defect group showed an obvious shrinkage (Supplementary Fig. 5a). Gross morphology of neotissues at 8 weeks revealed that the Defect group exhibited visible adhesion, swelling, and length contraction. In marked contrast, the ACF- and ACF-rP-regenerated tendons were compact; in particular, the ACF-rP-regenerated tendons possessed a much smoother surface and more evenly distributed structure (Supplementary Fig. 5b). The results of histological analysis further confirmed better regenerative outcomes with rPOSTN. Initially, abundant cells were recruited in the experimental groups, with more cells residing along the scaffold longitudinal axis in the ACF-rP group; while fractures and vacuoles were formed in the Defect group. At 4 weeks, the ACF and ACF-rP groups showed obvious ingrowth of tendon matrix at differing degrees of maturation and gradual reduction of inflammatory cell infiltration. Notably, massive spindle-shaped, tenocyte-like cells and aligned collagen fibrous structures were present in the ACF-rP group (Fig. 6b). After 8 weeks of healing and remodeling, the inflammation subsided considerably. The ACF-rP group achieved a more satisfactory regeneration outcome than the ACF group, as indicated by the more compact and orderly alignment of ECM deposition similar to native tendons (Fig. 6b). Cross-sections of the regenerated tendons also showed better collagen matrix deposition and remodeling in the ACF-rP group. Without scaffolds to maintain the continuity of the Achilles apparatus, obvious heterotopic ossification was observed with hypertrophic chondrocytes and bone marrow-like areas in the Defect group (Supplementary Fig. 5c). These observations further suggested that the biomimetic ACF provides favorable biomechanical cues to guide tendon regeneration.

To identify the cells and molecules involved in modulation of tendon regeneration and repair, immunofluorescence staining was performed for stem cell and tendon-specific markers. In the early stage of tendon healing, more endogenous CD146^+^ stem cells were recruited by rPOSTN to the defect area (Fig. 6c). An initial burst release might contribute to promoting the recruitment of endogenous stem cells at the injury site^5^. This finding was further confirmed by the results of Transwell assays in vitro, whereby addition of exogenous rPOSTN induced additional TSPC migration (Supplementary Fig. 6a). Moreover, the CD146^+^ stem cells showed higher expression levels of Sox2 and Oct4 in the ACF-rP groups than in the other groups, which was consistent with our in vitro findings that TSPC stemness could be modulated by rPOSTN (Fig. 6c). At 2 weeks postoperatively, the ACF-rP group showed much higher expression of Postn, compared to the other groups (Supplementary Fig. 6b). In the middle stage, Scx, Mkx, Col, and Tnmd were expressed at higher levels in the ACF-rP group, compared to the other groups (Supplementary Figs. 5d, 6c, d). This might be caused by the proliferation and differentiation of recruited endogenous TSPCs induced by rPOSTN in vivo. Unexpectedly, the ACF-rP regenerated neotendons exhibited more blood vessels along with more abundant CD31 and VEGFR2 expression, relative to the other groups (Supplementary Fig. 6d). The interconnected porosity of ACF might be conducive to cell and vessel infiltration, and rPOSTN might further promote the angiogenesis via the TSPC secretome. At a later stage, delivery of rPOSTN yielded more mature tendon-specific ECM, as reflected by high expression levels of tendon-associated proteins Tnc and Tnmd (Fig. 6d).

To determine that the newly formed tissues were not scar or fibrocartilaginous tissues, Alcian Blue and Sirius Red stainings were performed for tissue sections at 8 weeks postoperatively. Whereas the tissues from the Defect group exhibited strongly positive staining, neotissues from the ACF and ACF-rp groups were almost negative for Alcian Blue staining, indicating that newly formed tissues were not fibrocartilaginous tissues. Sirius Red staining showed that neotissues from the ACF and ACF-rp groups presented a bright red in color and continuously oriented morphology, whereas the tissues from the Defect group exhibited relatively irregular arrangement (Supplementary Fig. 7a). Furthermore, immunofluorescence staining of fibrocartilaginous markers Col2 and Col3 showed that there was almost no positive expression in neotissues from the ACF and ACF-rp groups, whereas results of the Defect group presented large amount of areas with positive expression (Supplementary Fig. 7b, d). To further confirm our results, we performed immunofluorescence staining of tissue sections at 4 and 8 weeks postoperatively with the common scar tissue markers, including S100A4, α-SMA, and CD68, and found that both the ACF and ACF-rp groups showed no evident expression (Supplementary Fig. 7b–d). These data all indicated that neotissues from the ACF and ACF-rp groups were not scar or fibrocartilaginous tissues.

Postn could regulate collagen fibrillogenesis and the biomechanical properties of connective tissues, and promote collagen cross-linking by reinforcing the proteolytic activation of lysyl oxidase^50,51^. The aligned pattern and diameter of collagen fibrils are predictors of mechanical properties and healing of tendons^4,11^. To confirm the relationships between these factors and neotendon formation, we investigated the ultrastructure of the neotendon by SEM and TEM. SEM revealed that delivery of rPOSTN yielded ECM with greater density and more parallel arrangement at both 4 and 8 weeks postoperatively (Fig. 6e, Supplementary Fig. 5e). At the nanoscale level, more mature aligned collagen fibrils formed with the fibrillogenesis from 4 weeks to 8 weeks postoperatively in both the ACF and ACF-rP groups. Specifically, delivery of rPOSTN yielded more mature collagen fibrils at both time points. At 4 weeks postoperatively, microfibrils (<100 nm) were dominant (~92.9%) in the ACF group, while ~43% fibrils (100–200 nm) were formed by ACF-rP (Supplementary Fig. 5e). At 8 weeks postoperatively, mature fibrils were dominant (~72%) in the ACF-rP group, while only ~20% fibrils were detected in the ACF group. In contrast, the Defect group consisted of mainly disorganized small collagen microfibrils. Analysis of circularity, as a measure of collagen alignment, further showed that the circularity of the ACF-rp group was significantly higher than that of the ACF group, and much closer to that of native tendons (Fig. 6e).

**Fig. 7:**
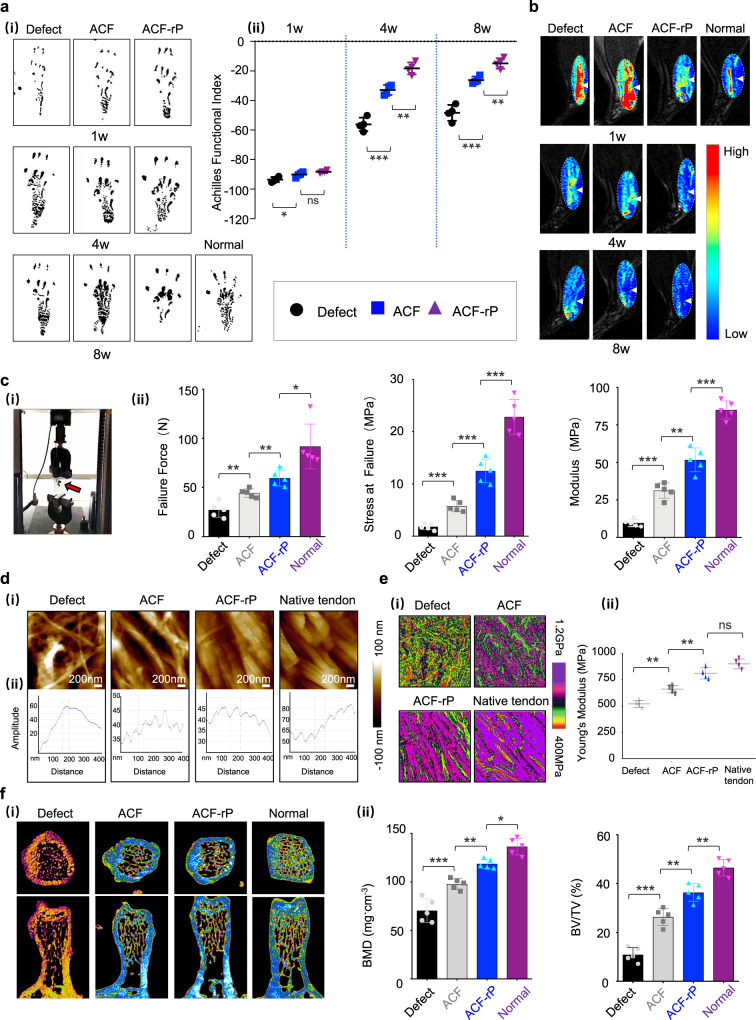
Functional regeneration and repair of injured tendons using ACF loaded with rPOSTN. **a** (i) Footprints of normal and experimental rats from the Defect (without implants), ACF and ACF-rP groups at 1, 4, and 8 weeks postoperatively. (ii) Semi-quantification of AFI (*n* = 4 rats per group, by one-way ANOVA with Tukey’s post hoc test: ***P < 0.001, **P < 0.01, *P < 0.05, ns: not significant). **b** T2-weighted MRI scans of regenerated Achilles tendons of each group at 1, 4, and 8 weeks postoperatively (*n* = 5 rats per group). White arrows: Achilles tendons. **c** (i) Images of dynamometer and (ii) Biomechanical properties of the regenerated tendons (failure force, stress at failure, and modulus) of each group at 8 weeks postoperatively (*n* = 5 rats per group, by two-tailed Student’s *t* test: ***P < 0.001, **P < 0.01, *P < 0.05). **d** (i) AFM morphology of native tendon and newly formed collagen fibrils of each group at 8 weeks postoperatively. (ii) Section analysis of single collagen fibrils in (i). **e** (i) Corresponding AFM property maps of native tendon and newly formed collagen fibrils of each group at postoperative week 8 in (d). (ii) Semi-quantification of Young’s modulus in (i) (*n* = 5 rats per group, by one-way ANOVA with Tukey’s post hoc test: **P < 0.01, ns: not significant). **f** (i) Micro-CT images of trabecular bone volume in calcaneus head of each group at 8 weeks postoperatively. (ii) Semi-quantification of BMD and BV/TV in (i) (*n* = 5 rats per group, by one-way ANOVA with Tukey’s post hoc test: ***P < 0.001, **P < 0.01, *P < 0.05). Data are represented as mean ± SD. Exact *P* values were given in the Source Data file.

To evaluate the functional performance of healing tendons, the Achilles Functional Index (AFI) was documented at 1, 4, and 8 weeks postoperatively. The AFI of normal rats is near zero; a more negative AFI value represents more serious hypomotility^52^. As shown in Fig. 7a, the hind paw prints of rats on the injured side in all groups were distinctly longer and narrower than those on the normal side at 1 week postoperatively; they showed a partial recovery at 4 weeks postoperatively. The AFI value based on the prints at 1 week postoperatively ranged from −95.5 to −87.2; it did not significantly differ between the ACF and ACF-rP groups. All AFI values increased gradually with the increasing healing time; the ACF-rP groups showed markedly higher values than the ACF and Defect groups. At 8 weeks postoperatively, the AFI value of the ACF-rP group approached the normal level (−14.98 ± 3.52), indicating excellent repair performance (Fig. 7a). This was further confirmed by videos documenting the condition of the ACF-rP restored rats; they could perform activities, such as standing and walking, without the use of any external fixation devices (Supplementary Movie 1).

Magnetic resonance imaging (MRI) combined with sagittal T2-weighted imaging was used for further noninvasive assessment of the healing dynamic changes and regeneration of Achilles tendons. The healthy Achilles tendon showed a low-intensity and homogeneous signal with smooth and clear contours in the hindlimb (Fig. 7b). At 1 week postoperatively, marked swelling, irregular contours, and heterogeneous high- intensity or discontinuous signals were observed, indicating successful establishment of the Achilles tendon defect and occurrence of inflammation in the defect region. At 4 weeks postoperatively, swelling remained but had subsided slightly; and the high-intensity signal regions also decreased gradually in all groups, especially in the ACF and ACF-rP groups. These observations indicated the positive regulatory role of the biomimetic ACF on reduction of the inflammatory response. At 8 weeks postoperatively, regions of high signal intensity and visible swelling were present in the Defect group, while marked reductions in the signal intensity were detected in the ACF and ACF-rP groups. Notably, the signal intensity, degree of swelling, and tissue contours showed the most significant improvements in the ACF-rP group; they became comparable to the characteristics of normal Achilles tendons.

Tendons possess a high elastic modulus under tension, but collapse under compression. Upon mechanical testing of the 8-week tendons using an Instron tension/compression system, we found that the failure force was much higher in ACF-rP-regenerated tendons (59.55 ± 8.09 N) than in the Defect group (27.22 ± 7.56 N) and ACF group (44.32 ± 4.32 N). rPOSTN delivery yielded regenerated tendons with an elastic modulus of 51.71 ± 8.01 MPa, which was near the value for native tendons (85.05 ± 6.03 MPa) and significantly higher than the values of tendons treated with ACF alone or without scaffolds (Fig. 7c). Other mechanical parameters including stiffness, work to failure and hysteresis showed the ACF-rp-regenerated neotendons possessed better mechanical properties (Supplementary Table 2). To assess the relationship between fibril pattern and tendon mechanics, atomic force microscopy (AFM) was performed to examine the fibrillar crimp and Young’s modulus of the regenerated tendons in situ. rPOSTN delivery produced typical collagen ultrastructure with native periodic crimp pattern of ~67 nm and higher Young’s modulus of 811.66 ± 54.74 MPa, compared to the Defect and ACF groups; the nanostructure and nanomechanics of the ACF-rP regenerated tendon were similar to those of the native tendon (Fig. 7d, e). These results suggested that rPOSTN is necessary and sufficient for regenerating tendons with tensile mechanical properties restoring similar to the native level. It should be noted that training exercises at later stage might be another factor to improve the mechanical recovery of the regenerated tendons.

The densities of cortical and trabecular bone in the humeral head are sensitive to structural and functional loss of the rotator cuff^53^. Therefore, we evaluated the bone quality of the calcaneus head that functions in the insertion of the regenerated Achilles tendons (Fig. 7f). Micro-computed tomography (micro-CT) images indicated obvious trabecular and cortical bone mass loss at 8 weeks postoperatively in the Defect group, compared to the other groups. In contrast, bone loss was markedly mitigated in the ACF and ACF-rP groups. Quantitative analysis indicated that the most remarkable loss occurred in the Defect group; bone mineral density (BMD) fell to 70.36 ± 12.07 mg cm^−3^ and bone volume over total volume (BV/TV) declining to 10.90%  ± 2.87%. However, the degree of reduction was significantly smaller in the ACF group, with BMD of 97.89 ± 5.63 cm^−3^ in and BV/TV of 26.32% ± 3.53%. Notably, values were significantly higher in the ACF-rP group (BMD, 118.75 ± 4.77 mg cm^−3^; BV/TV, 36.38% ± 3.63%) than in the ACF group, and were closer to those of normal tendons.

To confirm better healing outcomes by the delivery of rPOSTN in the longer term, we further performed experimental examinations of newly formed tissues at 12 weeks postoperatively, including histological staining, immunofluorescence staining of tenogenic and fibrocartilage markers and collagen ultrastructure. HE, Masson’s trichrome, and Sirius Red stainings showed that neotissues from the Defect group were sparse fibrocartilage-like tissues, while the ACF- and ACF-rp-regenerated neotendons exhibited more compact and better collagen matrix deposition compared to results at 8 weeks postoperatively. Notably, compared to the ACF group, the newly formed collagen bundles were more mature and orderly aligned, which might be attributed to the improvement of tendon functions (Supplementary Fig. 8a). This was further confirmed by the immunofluorescence staining of tenogenic markers Tnmd and Tnc. Strongly positive staining was found in both the ACF and ACF-rp groups; and the ACF-rp-regenerated neotendons exhibited more compact and orderly aligned collagen along with more abundant Tnmd and Tnc expression, relative to the ACF group (Supplementary Fig. 8b). To determine that the neotissue was not a scar or fibrocartilaginous tissue, Alcian Blue staining and immunofluorescence staining of S100A4 and Col2 were performed (Supplementary Fig. 8c, d). Both stainings confirmed that fibrocartilaginous tissues were formed in the Defect group, while neotissues from the ACF and ACF-rp groups didn’t advance into fibrocartilaginous tissues. Since collagen alignment and diameter are preditors of the biomechanical properties of tendons^4,11^, we investigated the ultrastructure of neotissues by SEM and TEM (Supplementary Fig. 8e). Consistent with the microstructure examination by histological staining, the delivery of rPOSTN yielded more orderly aligned collagen matrix with greater density, diameter and circularity, relative to the ACF group, indicating higher biomechanics of the ACF-rp-regenerated neotendons.

## Discussion

**Fig. 8:**
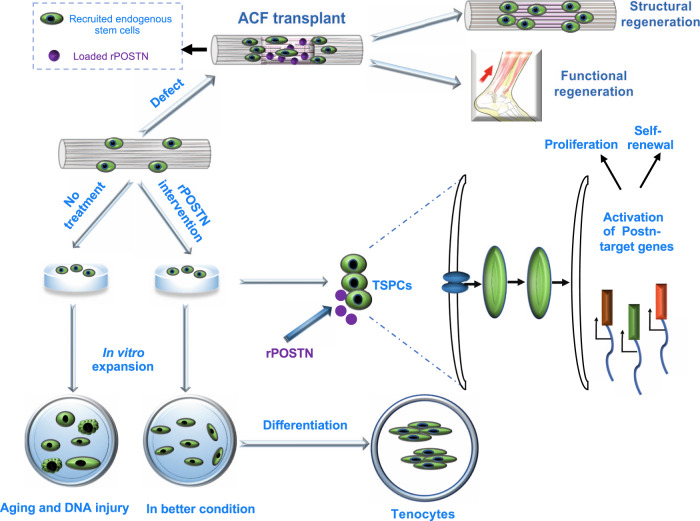
The potential role of Postn in regulating TSPC functions and tendon regeneration. Schematic illustration shows that rPOSTN modulates TSPC multiple cellular processes, including proliferation, stemness, anti-senescence and tenogenic differentiation in vitro, and boosts structural and functional tendon regeneration by a combination of biomimetic parallel-aligned collagen scaffolds in vivo.

Chronic and acute tendon injuries or defects are very common, and functional tendon generation remains a major challenge in regenerative medicine^1,3^. Here, we presented a functional tendon regeneration strategy in the acutely injured model by jointly harnessing of the regenerative potential of a specific protein (Postn) involved in postnatal tendon development and a biomimetic parallel-aligned collagen scaffold ACF. rPOSTN treatment could maintain TSPC stemness and promote their tenogenic differentiation potentials in vitro. And Postn knockdown by siRNA interference reduced TSPC multiple biological functions. The rare TSPC population was successfully enriched by rPOSTN treatment, then underwent tenogenic differentiation leading to tendon regeneration. By mimicking the tendon fibril micro/nanopattern, ACF provided an optimal microenvironment for TSPC growth and differentiation. The ACF-rP-regenerated tendons showed near-restoration of native-like mechanical properties, collagen micro/nanostructure and orientation, and locomotion functions (Fig. 8). Further studies would be needed to explore the ACF-rp-mediated regeneration strategy for chronically pathological tendons.

Existing physicochemical strategies to encourage tendon repair or expand TSPCs in vitro include hypoxia, three-dimensional culture, substrate patterning and delivery of common growth factors, such as TGF-β, IGF-1, PDGF, and GDF5. However, long-term maintenance of TSPC stemness and functional regeneration of tendons have not been achieved^7–11^. Using transcriptional profiling of postnatal tendon developmental processes, we showed that Postn promoted self-renewal and differentiation capacity of TSPCs during early passage or after long-term passage by activating two core stemness-related transcription factors, Sox2 and Oct4. Similarly, it has been shown that Postn from periosteum regulated stemness of skeletal stem cells and contributed to bone regeneration^14^. Recent evidence have shown that transient activation or coordination of several nuclear reprogramming factors is beneficial for the amelioration of stem cell aging and enhancement of regenerative ability^54^. Similarly, treatment with rPOSTN could slow cell senescence and DNA injury in both replicative and stress-induced premature senescence. rPOSTN-treated TSPCs had superior spheroid-forming capacity and were capable of regenerating a tendon ECM structure. Currently, spheroid and organoid modes are widely used in stem cell cultivation and disease models for drug screening^29–31,55^. Hence, this unexpected finding may contribute to optimization of methods for TSPC culture, as well as to the discovery of more key genes that promote TSPC function, and tendon regeneration and repair.

With regard to regeneration of the full-cut tendon defect, it is crucial to utilize the suitable biomaterials to load exogenous growth factors and provide a mechanical support for recruitment of endogenous stem cells or fibroblastic cells, promote natural cell–cell communication, and maintain ECM integrity in the early stage of tendon regeneration^5,56–58^. Currently available scaffolds (e.g., autografts, allografts, xenografts, and synthetic biomaterials) have several inherent limitations, including donor site morbidity, poor graft integration, high rates of recurrent tearing, and inflammatory responses; these limitations can ultimately cause failed integration of biomechanical and structural tendon regeneration^42,59,60^. To mimic the native tendon physicochemical architecture, we developed a dynamic diffusion-template self-assembly strategy that allowed orderly assembly of parallel-aligned ECM collagen fibrils. Similar to native tendons, the assembled ECM fibrils could provide an optimal microenvironment for TSPC growth and tenogenic differentiation in vitro and in vivo. Moreover, heterotopic ossification is a chief factor that prevents functional recovery in tendon tissue engineering^61,62^, which did not occur in the groups treated with ACF in the present study. This finding confirmed that ACF, mimicking natural tendon’s parallel-aligned collagen structure, is conducive to tenogenic differentiation and avoids undesirable bone formation in vivo. The regeneration outcomes here were comparable to previous studies with use of collagen scaffolds^44,52^. To maximize the regeneration efficiency, further efforts would be made to improve and optimize ACF to realize personalized and lifelike tendon scaffolds in microarchitecture.

Tissue engineering often resorts to cells, biomaterials, and signaling molecules; and various cells have been applied for ex vivo culture and cell transplantation^63,64^. In contrast, recruitment of endogenous progenitors or “cell homing” is a more effective method for repairing and regenerating certain tissues and organs, thereby avoiding the need for tedious and poorly developed cell expansion methods^65–67^. It has been demonstrated that Postn, as a matrixcellular protein, modulate cell–cell and cell–matrix communication^19^. Excellent works by Noack et al. have shown that Postn secreted by mesenchymal stem cells promoted tendon-like tissue formation by regulating ECM production in an ectopic transplantation model. This foundational finding provides inspirational insights that Postn may exert crucial biological functions in resemblance to two previously proved vital tendon-related ECM proteins biglycan and fibromodulin^2,68^. Based on those studies, we explored the role of ECM protein Postn in tendon development, and regeneration and repair. By analyzing transcriptional profiling of tendon developmental processes, we singled out the ECM protein Postn, and revealed its biological functions, including modulation of TSPC stemness, tenogenic capacity, and delay of TSPC senescence. Several studies have reported that Postn could regulate tissue repair and regeneration in other organs. For example, Postn could induce reentry of differentiated cardiomyocytes into the cell cycle to enhance regenerative capacity of adult hearts^34^. As to periodontal regeneration, Postn could promote the migration of host cells and collagen fibrillogenesis to boost regeneration^69^. Moreover, it has been shown that Postn could promote tissue healing process in skin and cardiac tissues by modulating the inflammatory balance^70,71^. In the present study, transplantation of ACF with rPOSTN-restored TSPCs into nude mice regenerated tendon-like tissues, which exhibited a histological and physical structure, and specific tenogenic fate similar to that of native tendons. More importantly, delivery of rPOSTN proteins recruited more endogenous TSPCs, promoted recruited TSPC proliferation, and enabled better structurally and functionally endogenous regeneration in the full-cut tendon defect model without cell transplantation. It should be noted that rPOSTN might have an effect on other cells, including immune cells, tendon fibroblast cells, and potentially existing tendon fibroadiopgenic progenitors. For example, a previous work found that delivery of connective tissue growth factor in vivo also influenced CD146^−^ tendon cells besides CD146^+^ stem/progenitor cells, which in turn produced a paracrine effect on the CD146^+^ stem/progenitor cells^5^. This phenomenon might exist in the Postn-mediated tendon regeneration, which would be investigated in our future work.

In conclusion, our data suggest that structural and functional tendon regeneration and repair by rPOSTN stimulation of TSPC stemness and differentiation may represent a simple and translatable therapeutic strategy that avoids difficulties associated with cell transplantation and the complexity of delivery system.

## Methods

### Ethics statement

All experimental procedures used in this study were performed in compliance with animal welfare ethical regulations and approved by the Animal Use and Care Committee of Peking University (LA2018302). Male Sprague Dawley neonatal (1 day, body weight of 1.5–2 g) and 6–8-week-old rats (body weight of 200–250 g) were obtained from Weitong Lihua Experimental Animal Center (China). Animals were housed in ventilated cages on a 12:12-hour light/dark cycle with ad libitum access to food and water. During the different experiments, rats and nude mice were removed if they died prematurely or suffered from tumors. All animals were fed a normal diet and euthanatized in a fed state by cervical vertebra dislocation.

### Gene expression profiling by microarray

Tendon tissues from the neonatal and 6–8-week-old rats were harvested and total RNA was extracted using Trizol reagents (Thermo Fisher Scientific) and purified with an RNeasy mini kit (Qiagen). Biotinylated cDNA was prepared according to the standard Affymetrix protocol from 150 ng total RNA by using Ambion® WT Expression Kit. Following labeling, fragmented cDNA was hybridized for 16 h at 45 °C on Affymetrix Rat Transcriptome Array 1.0 [transcript (gene) CSV version]. GeneChips were washed and stained in the Affymetrix Fluidics Station 450. All arrays were scanned by using Affymetrix® GeneChip Command Console, which was installed in GeneChip® Scanner 3000 7 G. The gene expression data were analyzed with robust multichip analysis (RMA) algorithm using Affymetrix default analysis settings and global scaling as a normalization method. Values presented are log2 RMA signal intensity. The microarray data are deposited at NCBI Gene Expression Omnibus (GSE158342). Differentially expressed genes were identified based on the Student’s *t* test for comparison of the two groups. The threshold set for up- and down-regulated genes was a fold change > 2.0 and a *P* value < 0.05.

### Cell isolation and expansion

Primary TSPCs were isolated from neonatal and 6–8-week-old rats according to the previously established procedure^2^. Briefly, the harvested Achilles tendon was minced and digested fully in 3 mg/ml Type I collagenase (Thermo Fisher Scientific) and 4 mg/ml dispase (Roche) at 37 °C for 30 min. After filtrating through a 70-μm strainer, single-cell suspensions were cultured in low-glucose Dulbecco’s modified Eagle’s medium (DMEM, Hyclone) supplemented with 20% fetal bovine serum (FBS, Thermo Fisher Scientific), 2 mM L-glutamine (Thermo Fisher Scientific), and 100 U/ml penicillin/streptomycin (Thermo Fisher Scientific). At 80%–90% confluence, cells were trypsinized, centrifuged, resuspended in growth medium as passage 1 cells, and incubated in 5% CO_2_ at 37 °C, with fresh medium changing every 2–3 days.

### Monoclonal selection

Majority of the cultured cells presented fibroblast-like after two or three passages and then were trypsinized, resuspended, and seeded at very low density (2–4 cells/cm^2^) in 96-well plates with about 100 μl medium. After 7 days, colonies were detected by microscopy and marked carefully. Then, the plates were gently rinsed by PBS solution twice and detached carefully by Trypsin-EDTA Solution. Selected colonies were sub-cultured into 12-well plate and then identified by flow cytometry.

### Flow cytometry

We incubated 1 × 10^6^ TSPCs (isolated from 6–8-week-old rats), respectively, with primary antibodies of CD90, CD105, CD44, CD45, and CD34 for 1 h and fluorescent secondary antibody for 1 h at 4 °C, then analyzed the samples using flow cytometer (BD Acuuri C6) to calculate the expression of cell surface markers in isolated TSPCs (Supplementary Fig. 9).

### siRNA knockdown

To knockdown Postn expression in TSPCs isolated from 6–8-week-old rats, siRNA transfection was performed according to the manufacturer’s instructions. Fluorescein-conjugated control siRNA was used as a control and as a method of evaluating transfection efficiency. All siRNA products were purchased from GenePharma Company (China). The RNAi oligonucleotide sequences used for *Postn* knockdown were: 5ʹ-UUUAAUGACUGGUUCUCCGTT-3ʹ.

### Cell proliferation and CFU-F assays

Cell proliferation was evaluated by examining Ki67 expression by immunofluorescence staining after undergoing corresponding treatment. For CFU-F assay, single-cell suspensions of TSPCs (1000 cells/well, isolated from 6–8-week-old rats) were seeded and incubated in six-well plates for 12–14 days in the growth medium and fixed with 4% paraformaldehyde (PFA) (Sigma-Aldrich). Then, 0.1% crystal violet solution (Solarbio) was used to stain the cells. Colonies of >30–50 cells were defined as single colony unit, and the number of clusters was counted using Image J 1.52a software.

### Cell migration

Cell migration was evaluated using 8-µm pore size Transwell (Corning). The lower chamber was overlaid with different concentrations of rPOSTN (R&D System) supplied by 1% FBS in 750 µl of DMEM. A total of 1 × 10^4^ TSPCs were cultured in 500 μl of DMEM medium containing 1% FBS with undergoing serum starvation for 12 h and then transferred in the Transwell insert. After 16 h, unmigrated TSPCs were removed, and migrated TSPCs were washed gently with PBS, fixed with 4% PFA, stained by 0.1% crystal violet and counted using Image J 1.52a software.

### In vitro model of oxidative stress injury and senescence staining

TSPCs were pretreated with 100 ng/ml rPOSTN protein or transfected with siRNA. To mimic the oxidative stress microenvironment, TSPCs were exposed to 200 μM H_2_O_2_ (Sigma-Aldrich) in serum-free DMEM for 4 h. Anti-oxidative stress capacity of cells was detected by immunofluorescence staining of γ-H2AX and SAβ-gal staining according to manufacturer instructions (Cell Signaling Technology). For the detection of P53, P21, and γ-H2AX, protein lysates were prepared and detected by Western blotting (Supplementary Tables 3, 4).

### Multipotent differentiation

With regard to the differentiation experiment, TSPCs were cultured in 6-well plates (50,000 cells/well). Osteogenic, adipogenic, chondrogenic, and tenogenic differentiation were induced by a corresponding differentiation medium. The osteogenic differentiation medium contained a growth medium supplemented with 10 nM dexamethasone (Sigma-Aldrich), 5 mM β-glycerophosphate (APEXBIO), and 0.05 mM l-ascorbic acid 2-phosphate (Sigma-Aldrich). The adipogenic differentiation medium consisted of a growth medium supplemented with with 500 μM isobutyl-methylxanthine, 60 μM indomethacin (Sigma-Aldrich), 0.5 μM hydrocortisone, and 10 μM insulin (Sigma-Aldrich). The chondrogenic differentiation medium was a Gibco StemPro chondrogenic differentiation kit (Thermo Fisher Scientific). The tenogenic differentiation medium contained a growth medium supplemented with 10 ng/ml TGF-β1 (Peprotech), 10 ng/ml GDF-5 (R&D System), 0.05 mM l-ascorbic acid 2-phosphate. After a two-week induction, Alizarin Red S, Oil Red O, Toluidine Blue, Sirius Red and Masson’s trichrome stainings were applied to evaluate osteogenic, adipogenic, and tenogenic differentiation capacity, respectively.

### Three-dimensional spheroid formation assay

After reaching the 10th passage, single cells dissociated from the adherent monolayer of TSPCs were plated in Corning Costar Ultra-Low Attachment 6-well plates in serum-reduced DMEM supplemented with 10 ng/ml FGF2 (Peprotech), 20 ng/ml EGF (Peprotech). Formed spheroids were observed and counted under the microscope at day 7. Then, we transferred formed spheroids or scattering cells in the normal attachment 6-well plates with Matrigel (Corning), and induced tenogenic differentiation. HE, Sirius Red, and immunofluorescence stainings were applied to evaluate differentiation capacity. SEM and TEM were applied to examine micro/nanostructure of newly formed collagen or differentiated cells.

### RT-qPCR

Total RNA was isolated from the primary cells and tendon tissues using a Trizol reagent (Thermo Fisher Scientific) according to the manufacturer’s instruction. Then the mRNA was converted to complementary DNA by reverse transcriptases, and PCR was carried out using gene-specific primers and SYBR Green (Thermo Fisher Scientific) on 7900HT Fast Time PCR. The primers synthesized were given in the Supplementary Information (Supplementary Table 5).

### Western blotting

Total proteins from cell lysates and tissue homogenates were harvested by RIPA Buffer (Thermo Fisher Scientific) with Protease/Phosphatase Inhibitor Cocktail (Thermo Fisher Scientific). Cell lysate proteins were separated by 10% SDS–polyacrylamide gel electrophoresis, and then transferred to polyvinylidene difluoride membranes and blocked in 5% nonfat milk. The membranes were probed with corresponding primary antibodies overnight at 4 °C. Membranes were washed three times in TBS with 0.1% Tween-20 for 5–10 min each wash, and appropriate secondary antibodies were incubated for 1 h, washed twice in TBS with 0.1% Tween-20 and imaged. Detailed information of primary and secondary antibodies was listed in the Supplementary Table 3. The uncropped gel images were shown in Supplementary Figs. 10–12.

### Biomimetic parallel-aligned collagen fibril assembly

Based on our previously established protocol for collagen self-assembly^39,40^, hierarchical organization of ACF was achieved by the following adapted procedure: Type I tropocollagen solution from rat tails (Corning, 100 mg, 3–4 mg/ml, pH < 2) was continually injected into a dialysis bag (3500 Da), which was immersed in a solution containing 200 mM KCl, 30 mM Na_2_HPO_4_, and 10 mM KH_2_PO_4_ (pH = 7). After 24 h, the assembled collagen was rinsed with ddH_2_O for three times and crosslinked by using 1 wt% 1-ethyl-3- (3-dimethylaminopropyl)-carbodiimide in 80% ethanol for 4 h, washed in 1 wt% glycine solution and finally lyophilized for use. For mechanical tensile tests, ACF (2 mm in diameter) was immersed in PBS for 10–15 min, and examined using an Instron tension/compression system with a load cell of 100 N at a tensile speed 2 mm/min.

### Controlled release assay of rPOSTN from ACF in vitro

The controlled release assay of rPOSTN from ACF was performed as follows: 500 ng rPOSTN was added to ACF in a 24-well plate and lyophilized. Subsequently, 1 ml PBS (pH = 7.4) was added to each well, and then the plate was placed on a horizontal shaker with a speed of 80 rpm at 37 °C. The supernatant was collected after 0, 1, 2, 3, 5, 7, 10, 14, 18, 22, 26, 30 days. The concentration of rPOSTN was analyzed by a rat POSTN ELISA kit according to the manufactory’s instruction (Meimian, Jiangsu, China) at each time point, and corresponding sustained release curves were drawn.

### In vivo subcutaneously transplantation

TSPCs (1 × 10^6^ cells) with or without rPOSTN treatment were seeded on sterilized scaffolds, and then implanted subcutaneously in the dorsum of athymic mice (6–8 weeks, BLAB/c nude mice) under anesthesia. Implants from 8 weeks were retrieved, observed by microscope, weighed by microbalance, and then were fixed with 4% PFA. Paraffin sections at a thickness of 4 μm were harvested, and followed by HE, Masson’s trichrome, immunohistochemistry, and immunofluorescence stainings. Implants from 4 weeks were retrieved, digested and then expanded in vitro for immunofluorescence staining of tenogenic markers.

### Tendon injury and repair animal model

For partially incised tendon injury, the Achilles tendon of Sprague Dawley male 6–8-week-old rats was exposed by a lateral incision under general anesthesia. For each leg, a gap wound (2 mm in width) was created by use of micro-operating instruments. The wound was then irrigated and skin was sutured. After operation, rats were allowed free cage activity with unrestricted access to normal food and drink. Five rats were sacrificed for histological evaluation at 1 week postoperatively. The other five normal rats without surgery were used as control.

To create a full Achilles tendon defect, the central one-third of the Achilles tendon (~4 mm in width) was removed from the distal apex of the muscle to the insertion of the calcaneus. The operated rats were classified into 3 groups: (i) without scaffolds (Defect group); (ii) with ACF scaffold only (ACF group); (iii) ACF scaffold loaded with rPOSTN (ACF-rP group). The scaffold was inserted in the tendon defect and sutured to the broken ends using Ethicon 6-0 suture. After operation, surgical limbs were immobilized for 1 week. Upon sacrifice, limbs from each experimental group were used for histological evaluation, respectively, at 2, 4, 8, and 12 weeks.

To evaluate the healing outcomes of the Achilles tendons, their motility was assessed by Achilles functional index (AFI) at 1, 4, and 8 weeks postoperatively, adopting a modified method from a previous study^52^. Briefly, a restrictive roadway (100 cm long and 25 cm wide) was covered with a white paper. After their hind paws were evenly dipped with black ink, the rats were allowed to walk freely, printing black footprints on the white paper. To quantify the AFI of the rats, footprints were scanned (Epson Scanner, Japan). We defined and acquired related footprints’ parameters including print length (PL), toe spreading length (defined as the distance between the first and fifth toes, TS), and intermediary toe spreading length (defined as the distance between the second and fourth toes, IT). Then, according to the difference between the normal (N) and the experimental values (E), three footprints dimension factors including print length factor (PLF), toe spreading length factor (TSF), intermediary toe spreading length factor (ITF) could be acquired by use of the following equations:

1 $${\mathrm{PLF}} = ({\mathrm{NPL}} - {\mathrm{EPL}})/{\mathrm{EPL}}$$

2 $${\mathrm{TSF}} = ({\mathrm{ETS}} - {\mathrm{NTS}})/{\mathrm{NTS}}$$

3 $${\mathrm{ITF}} = ({\mathrm{EIT}} - {\mathrm{NIT}})/{\mathrm{NIT}}$$

Finally, the AFI was calculated according to an established equation^52^

4 $${\mathrm{AFI}} = 74\left( {{\mathrm{PLF}}} \right) + 161\left( {{\mathrm{TSF}}} \right) + 48\left( {{\mathrm{ITF}}} \right)-5$$

### Immunohistochemistry and immunofluorescence

The harvested samples were fixed immediately in 10% neutral buffered formalin for 24 h, dehydrated using a gradient alcohol, and embedded in paraffin blocks. Histological sections with 5 μm in thickness were prepared using a microtome. HE, Alcian Blue, Sirius Red, and Masson’s trichrome stainings were performed according to standard procedures to examine the general appearance of soft tissues. For semi-quantitative analysis of Alcian blue staining, we randomly chose four tissue sections from four different animals in each group; and three different subregions in each section were randomly selected. The protein expression in the tissues was examined using immunohistochemistry or immunofluorescence. For immunohistochemistry, tissue sections were deparaffinized, blocked and incubated with anti-Postn, anti-Scx, anti-Mkx, anti-Ki67, anti-Col1, and anti-Tnmd overnight at 4 °C, followed by washing and incubating with horseradish-peroxidase-conjugated secondary antibodies. For immunofluorescence staining, sections or cells were incubated with anti-Postn, anti-Sox2, anti-Oct4, anti-CD146, anti-CD105, anti-Ki67, anti-γ-H2AX, anti-Col1, anti-Tnc, Anti-Tnmd, anti-Mkx, anti-Scx, anti-CD31, anti-VEGFR2, anti-CD68, anti-Col2, anti-α-SMA, anti-S100A4, and anti-Col3. FITC-Phalloidin and anti-Tubulin immunofluorescence staining was applied to visualize the cytoskeleton. The detailed information on different antibodies was shown in Supplementary Table 3. Next, the sections were incubated with FITC or Rhodamine -conjugated secondary antibodies. Nuclei were counterstained with DAPI. Confocal microscopic images were acquired with a laser-scanning microscope (LSM 510, Zeiss, Germany), and then processed with LSM 5 Release 4.2 software.

### Magnetic resonance imaging

Rat Achilles tendons were performed MRI scan using a 8-channel surface coil at 3.0 T (Philips Ingenia, Philips Healthcare, Netherlands) at 10 days (denoted as 1 weeks), 4 and 8 weeks postoperatively for five rats from each group. Acquisition parameters were as follows: sagittal T2-weighted imaging (Turbo spin echo, repetition time = 3104 ms, echo time = 80 ms, field of view = 90 × 110 mm, matrix size = 384 × 384, slice thickness = 1 mm, interslice distance = 0.1 mm).

### Macroscopic evaluation of regenerated tendons

At 4 and 8 weeks postoperatively, rats were euthanized and the Achilles tendon was exposed fully and photographed using a camera (Nikon, Japan). Then, the full-length tendon complexes including partial gastrocnemius and calcaneus were harvested.

### Mechanical testing

The harvested Achilles complexes at 8 weeks were used for mechanical testing, which was performed by applying an Instron tension/compression system with Fast-Track software (Model 5969, Instron, Canton, MA). The hindlimbs were wrapped in gauze immersed in PBS solution and frozen at −80 °C for future testing. Before testing, the hindlimbs were gradually thawed at room temperature, and all surrounding soft tissues were rigorously removed. Measurements of the cross-sectional area of tendons were performed using alginate dental impression paste. The complex was then fixed to custom-made clamps using high-strength thread. After applying a preload of 0.1 N, each complex was cyclically elongated between 0 and 0.5 mm for 20 cycles at 5 mm/min in advance. Subsequently, the complex underwent a load to failure test at an elongation rate of 5 mm/min. The load–elongation behavior of the complex and failure modes were recorded. The structural properties of the complex were calculated by failure force (N), modulus (MPa), stress at failure (MPa), stiffness (N/mm), work to failure (J), hysteresis (MPa*mm/mm), and stress relaxation (%).

### Scanning electron microscope

The microstructure of scaffolds, cell sheets, and neotissues were examined using SEM (Hitachi SU8020, Japan) at 10 kV. The cell sheets and neotissues were pre-fixed in 2.5% glutaraldehyde in PBS (pH 7.4) at 4 °C for 12 h and washed three times with PBS. All the samples were dehydrated in a graded series of ethanol (50–100%), critical-point dried, and sputter-coated with gold for 2 min at 20 mA.

### Transmission electron microscopy

The ultrastructure of scaffolds, cell sheet, and neotissues were examined by TEM (Hitachi HT7700, Japan) at 100 kV. The samples were double-fixed with 1% osmium tetroxide (Sigma Aldrich), stained with lead citrate and uranyl acetate, and embedded in epoxy resin. Transverse and longitudinal ultrathin sections with 70–100 nm were prepared with a Leica ultramicrotome and placed on copper grids. Counting and measurement of collagen diameter from TEM images were processed by marking the fibrils individually in Image J 1.52a software.

### Micro-CT scanning

We harvested calcaneuses from euthanized rat and fixed them in 10% neutral buffered formalin for 24 h. Then the calcaneuses were scanned with a Skyscan 1174 micro-CT system (Bruker, Belgium) at a resolution of 10.21 μm. The acquired axial images were imported into a NRecon and CTvox software for visualization and analysis. We defined the regions of interest as the areas proximal to the growth plate in the calcaneuses, in order to include the secondary trabecular spongiosa. BMD and BV/TV of the interested region for each specimen were also calculated by using CTAn software.

### Atomic force microscope

The nanostructure and nanomechanical properties of regenerated tendons were tested using AFM (Dimension Icon, Bruker, USA) operated under the peak-force tapping mode with a 1.0 Hz scan rate and a 250 mV amplitude set point^39,40^. A TAP150A probe was used, and deflection sensitivity was calibrated on the sapphire model. Data were analyzed using a Nanoscope Analysis software 1.60. To calculate the Young’s modulus, three scans of representative areas were performed for each specimen. Each scan generated a mapping image with 256 × 256 resolution for the Young’s modulus.

### Statistical analysis

All values were presented as mean ± SD. Statistical analysis was performed using GraphPad Prism software version 8 for Mac. Statistically significant differences were assessed by unpaired two-tailed Student’s *t* test and one-way analysis of variance (ANOVA) with Turkey’s post hoc test. *P* < 0.05 was considered as statistically significant. The representative data reported were performed at least three independent experiments.

### Reporting summary

Further information on research design is available in the Nature Research Reporting Summary linked to this article.

## Supplementary information

Supplementary Information

Description of Additional Supplementary Files

Supplementary Movie 1

Reporting Summary

## Data Availability

The microarray data are deposited at NCBI Gene Expression Omnibus “GSE158342”. Other databases used in the study are Gene Ontology online database (http://geneontology.org/), KEGG (https://www.kegg.jp/), and GSEA online database (https://www.gsea-msigdb.org/gsea/index.jsp). All other data supporting the findings of this study are available within the article and its Supplementary information files or from the corresponding author upon reasonable request. Source data are provided with this paper. A reporting summary for this Article is available as a Supplementary Information file. Source data are provided with this paper.

## Source data

Source Data
