# Supplementary material for: Functional regeneration and repair of tendons using biomimetic scaffolds loaded with recombinant periostin
Source: Nat Commun. 2021 Feb 26;12:1293. doi: 10.1038/s41467-021-21545-1 (PMC7910464; doi:10.1038/s41467-021-21545-1)
Supplement: Supplementary file 1 — Supplementary Information [file 41467_2021_21545_MOESM1_ESM.docx]

**Supplementary Information**

**Functional regeneration and repair of tendons using biomimetic scaffolds loaded with recombinant periostin**

Yu Wang^1^, Shanshan Jin^1^, Dan Luo^2^, Danqing He^1^, Chunyan Shi^3^，Lisha Zhu^1^, Bo Guan^4^, Zixin Li^1^, Ting Zhang^1^, Yanheng Zhou^1^, Cun-Yu Wang^5^, Yan Liu^1*^

^1^Laboratory of Biomimetic Nanomaterials, Department of Orthodontics, Peking University School and Hospital of Stomatology, National Engineering Laboratory for Digital and Material Technology of Stomatology, Beijing Key Laboratory of Digital Stomatology, Beijing 100081, China.

^2^State Key Laboratory of Heavy Oil Processing, College of New Energy and Materials, Beijing Key Laboratory of Biogas Upgrading Utilization, China University of Petroleum (Beijing), Beijing 102249, China.

^3^Department of Radiology, Beijing Anzhen Hospital, Beijing Institute of Heart, Lung & Vascular Diseases, Capital Medical University, Beijing 100069, China.

^4^Beijing National Laboratory for Molecular Science, Institute of Chemistry, Chinese Academy of Sciences, Beijing 100190, China.

^5^Laboratory of Molecular Signaling, Division of Oral Biology and Medicine, School of Dentistry and Jonsson Comprehensive Cancer Center, University of California Los Angeles, Los Angeles, CA 90095, United States.

^*^Corresponding author E-mail: [orthoyan@bjmu.edu.cn](mailto:orthoyan@bjmu.edu.cn).

**Table of contents**

Supplementary Figure 1. Postn is highly expressed in postnatal tendon development and endogenous tendon injury repair.

Supplementary Figure 2. Postn promotes TSPC stemness and tenogenic differentiation potentials in early passage *in vitro*.

Supplementary Figure 3. rPOSTN modulates stemness and differentiation capacity of TSPCs through the PIK3-AKT axis.

Supplementary Figure 4. Facilitation of TSPC tenogenesis by biomimetic parallel-aligned collagen fibrils.

Supplementary Figure 5. Macro-, micro- and nano- structures of neotendons regenerated by ACF loaded with rPOSTN.

Supplementary Figure 6. rPOSTN facilitates endogenous stem cell recruitment and tenogenesis-specific marker expression.

Supplementary Figure 7. No scar or fibrocartilaginous tissues are formed in neotendons from the ACF and ACF-rp groups.

Supplementary Figure 8. Macro-, micro- and nano- structures of neotendons regenerated by ACF loaded with rPOSTN at 12 weeks postoperatively.

Supplementary Figure 9. Flow cytometry analysis strategy.

Supplementary Figure 10. Original scans of the blots in Fig. 1d, 2c, 2f, 3d, 3f and 3i.

Supplementary Figure 11. Original scans of the blots in Supplementary Fig. 2c, 2e, 2f, 2g and 2i.

Supplementary Figure 12. Original scans of the blots in Supplementary Fig. 3b, 3e, 3j and 6b.

Supplementary Table 1. Mechanical parameters of ACF.

Supplementary Table 2. Mechanical parameters of neotissues at 8 weeks postoperatively.

Supplementary Table 3. List of primary and secondary antibodies used in the study.

Supplementary Table 4. List of reagents or resources used in the study.

Supplementary Table 5. List of primers used in the study.

Supplementary Table 6. Software and Algorithms.

**
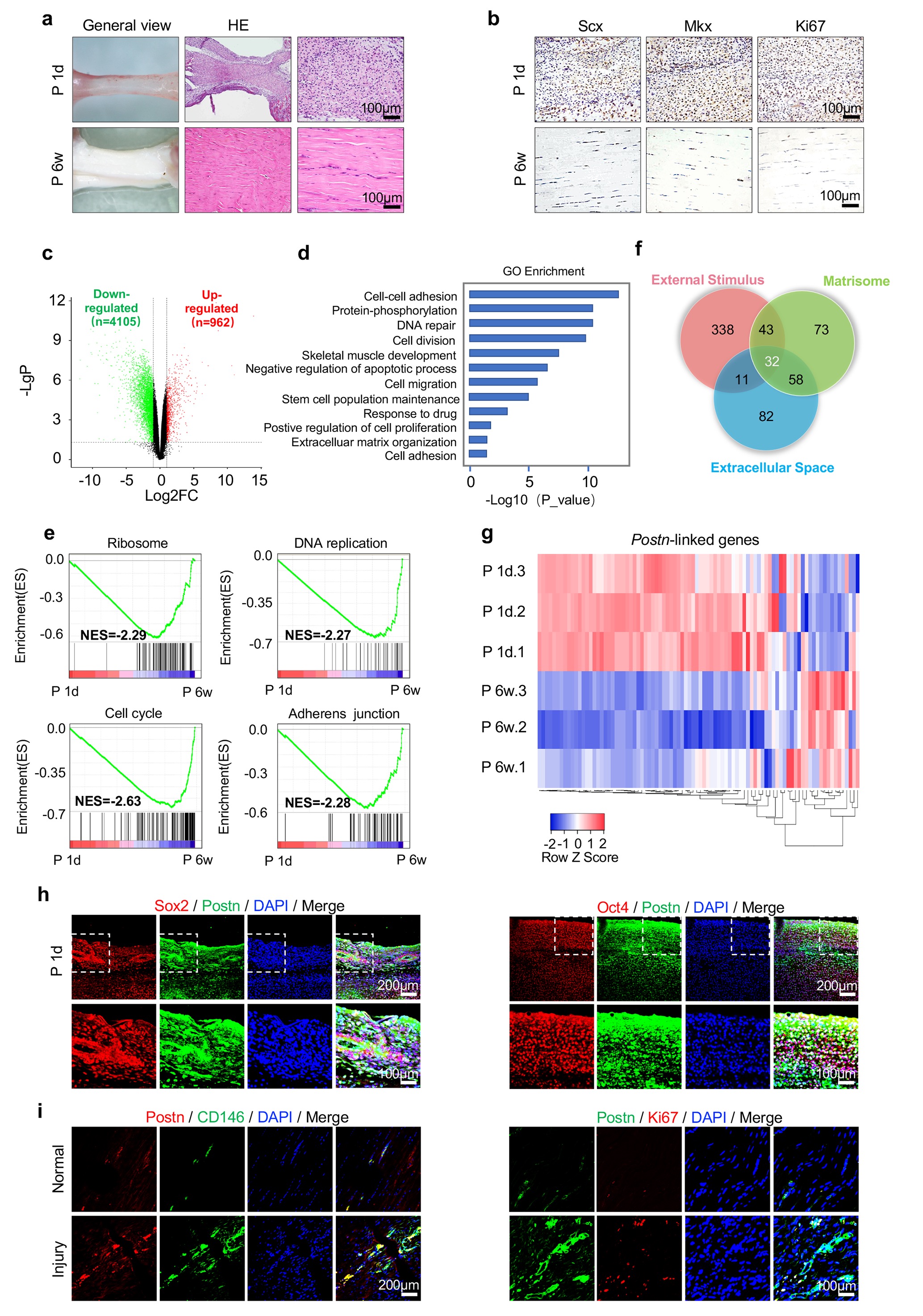
**

**Supplementary Figure 1. Postn is highly expressed in postnatal tendon development and endogenous tendon injury repair. a** Microscopic images and HE staining of the Achilles tendon from P 1d and P 6w rats. **b** Immunohistochemistry staining of Scx, Mkx and Ki67 in the rat Achilles tendons between P 1d and P 6w groups. **c** Volcano plot of gene expression array of postnatal tendon development profiling (*n* = 3 rats per group, by two-tailed Student’s *t*-test). **d** The enriched GO terms include cell−cell adhesion, cell division, and stem cell population maintenance (*n* = 3 rats per group)*.* **e** Gene set enrichment analysis of the regulated gene pathways with the KEGG database (*n =* 3 rats per group)*.* **f** Intersection of “external stimulus”, “extracellular space” and “matrisome” GO categories results in a list of 32 genes. **g** Heatmap of the 88 differentially expressed *Postn-*linked genes profiles between neonatal and adult tendons (*n =* 3 rats per group)*.* **h** Immunofluorescence staining of Postn, Sox2 and Oct4 in the sheath of 1-day tendons (*n =* 5 biologically independent samples). **i** Immunofluorescence staining of Postn, CD146 and Ki67 in normal and injured Achilles tendons at 1 week postoperatively (*n =* 5 biologically independent samples).

**
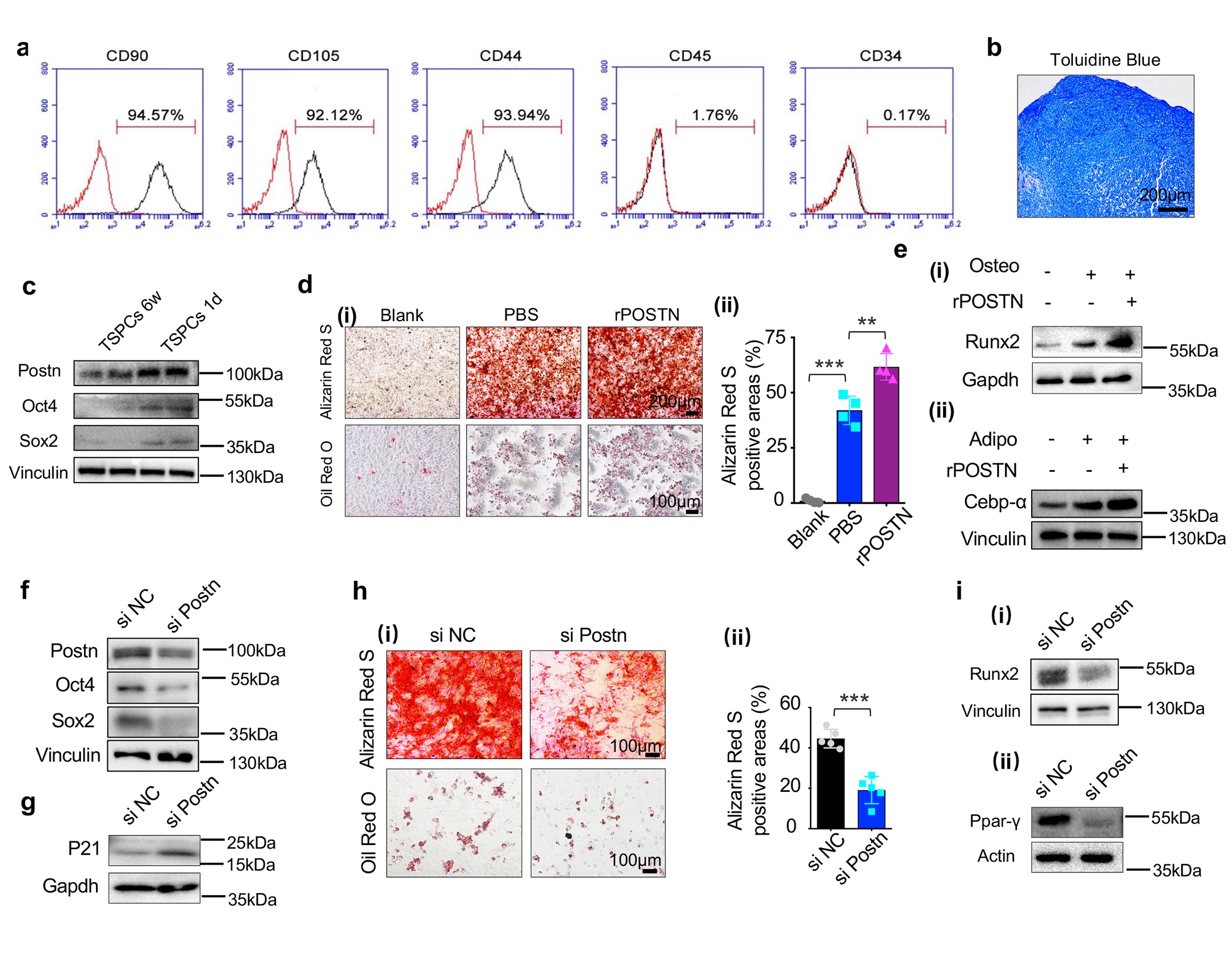
**

**Supplementary Figure 2.** **Postn promotes TSPC stemness and tenogenic differentiation potentials in early passage *in vitro*.** **a** Flow cytometry analysis of the expression of cell surface markers in TSPCs. **b** Toluidine blue staining of TSPCs after chondrogenic induction for 14 days. **c** Western blotting of Sox2 and Oct4 protein levels of P 1d and P 6w TSPCs (*n =* 3 biologically independent samples). **d** (i) Alizarin Red staining (upper panel) and Oil Red staining (lower panel) of PBS- and rPOSTN-treated TSPCs after induction for 14 days. (ii) Semi-quantification of (i). Blank: without induction medium (*n* = 4 biologically independent samples*,* by one-way ANOVA with Tukey’s post hoc test: *** *P* < 0.001, ** *P* < 0.01). **e** Western blotting of osteogenic gene Runx2 (i) and adipogenic gene Cebp-α (ii) protein levels of PBS- and rPOSTN-treated TSPCs. **f** Western blotting of Postn, Sox2 and Oct4 protein levels of si NC- and si Postn-treated TSPCs*.* **g** Western blotting of P21 protein levels of si NC- and si Postn-treated TSPCs suffering from H_2_O_2_ stimulation. **h** (i) Alizarin Red staining (upper panel) and Oil Red staining (lower panel) of si NC- and si Postn-treated TSPCs after induction for 14 days. (ii) Semi-quantification of (i) (*n* = 4 biologically independent samples, by two-tailed Student’s *t*-test: *** *P* < 0.001). **i** Western blotting of osteogenic gene Runx2 (i) and adipogenic gene Ppar-γ(ii) protein levels of si NC- and si Postn-treated TSPCs*.* Data are represented as mean ± SD. Exact *P* values were given in the Source Data file.


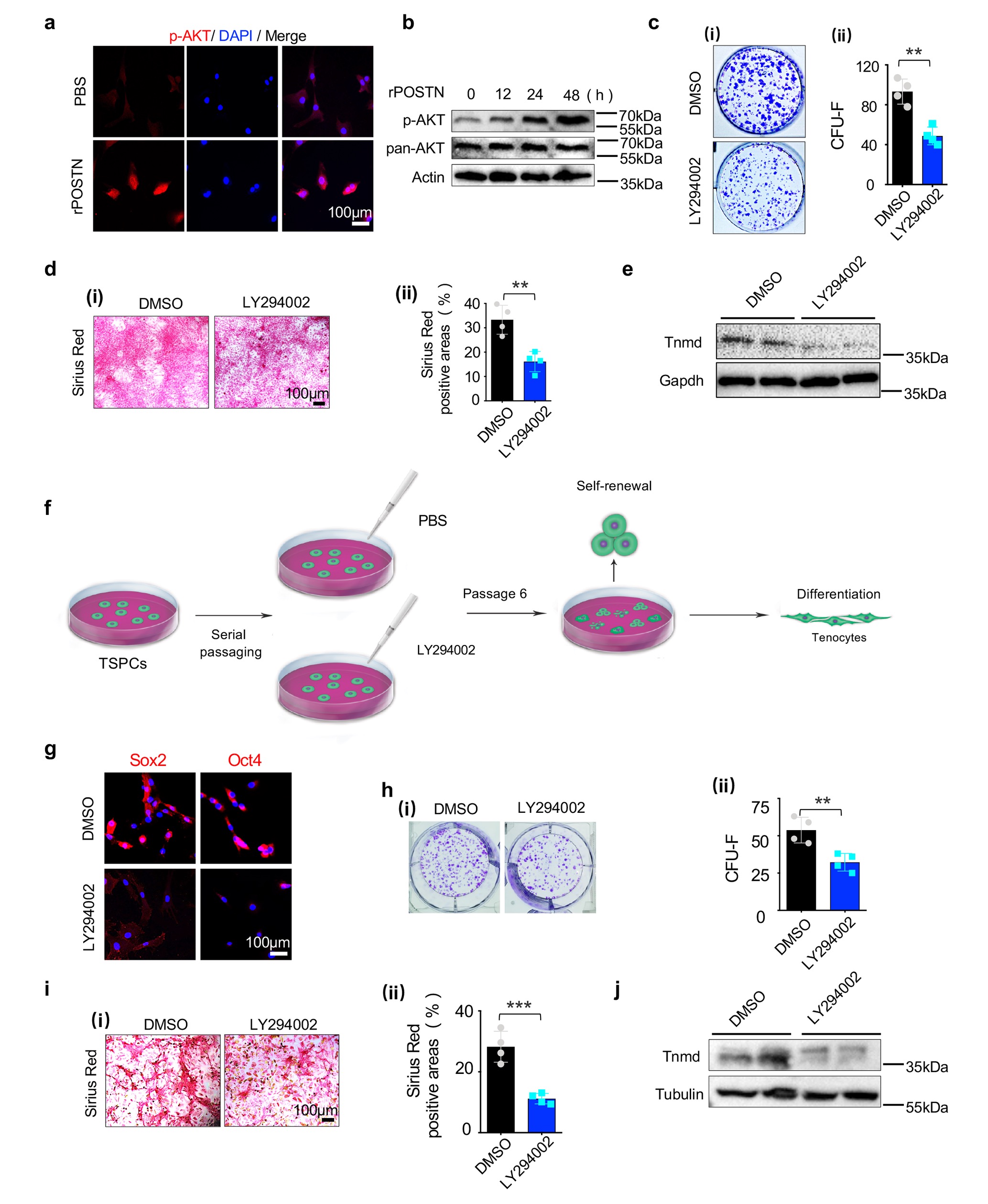


**Supplementary Figure 3. rPOSTN modulates stemness and differentiation capacity of TSPCs through the PIK3-AKT axis. a** Immunofluorescence staining of p-AKT of PBS- and rPOSTN-treated TSPCs. **b** Western blotting of p-AKT, pan-AKT protein expression in TSPCs after rPOSTN treatment at different concentrations. **c** CFU-F assay of DMSO- and LY294002-treated TSPCs (*n* = 4 biologically independent samples). **d** (i) Sirius Red staining of DMSO and LY294002-treated TSPCs in tenogenic differentiation medium (*n* = 4 biologically independent samples). **e** Western blotting of Tnmd protein levels of DMSO- and LY294002-treated TSPCs in tenogenic differentiation medium. **f** Schematic of the TSPC serial passaging with treatment of LY294002*.* **g** Immunofluorescence staining of Sox2, Oct4 of DMSO- and LY294002-treated TSPCs at 6th passage. **h** CFU-F assay of DMSO- and LY294002-treated TSPCs (*n* = 4 biologically independent samples). **i** (i) Sirius Red staining of DMSO and LY294002-treated TSPCs at 6th passage in tenogenic differentiation medium (*n* = 4 biologically independent samples). **j** Western blotting of Tnmd protein levels of DMSO- and LY294002-treated TSPCs in tenogenic differentiation medium. Data are represented as mean ± SD. Exact *P* values were calculated by two-tailed Student’s *t*-test and given in the Source Data file. *** *P* < 0.001, ** *P* < 0.01.

**
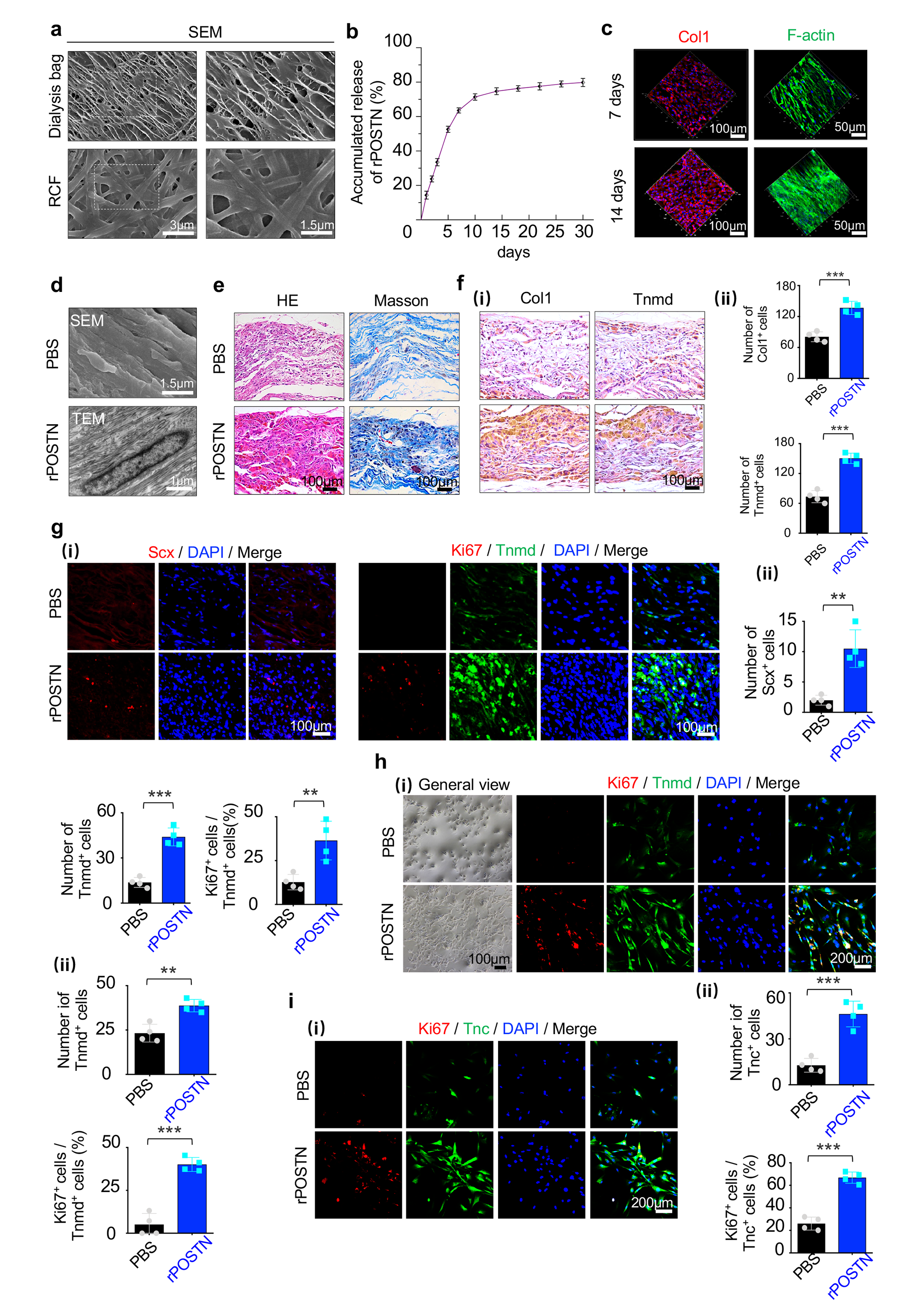
**

**Supplementary Figure 4. Facilitation of TSPC tenogenesis by biomimetic parallel-aligned collagen fibrils. a** SEM micrographs of dialysis bag and ACF. **b** The controlled release curve of rPOSTN from the ACF *in vitro*. **c** Three-dimensional immunofluorescence staining of Col1 and F-actin of TSPCs seeded on the ACF for 7 days and 14 days. **d** SEM and TEM of TSPCs seeded on the ACF for 14 days. **e** HE and Masson’s trichrome staining of cross sections of neotissues by implantation of ACF with PBS- and rPOSTN-treated TSPCs in the 10th generation after 8 weeks. **f** (i) Immunohistochemistry staining of Tnmd and Col1 of neotissues in **c**. (ii) Semi-quantification of (i) (*n* = 4 biologically independent samples). **g** (i) Immunofluorescence staining of Scx, Tnmd and Ki67 of neotissues. (ii) Semi-quantification of (i) (*n* = 4 biologically independent samples).  **h** (i) General view and immunofluorescence staining of Tnmd and Ki67 of cells isolated from neotissues after 4-week subcutaneous transplantation. (ii) Semi-quantification of (i) (*n* = 4 biologically independent samples). **i** (i) Immunofluorescence staining of Tnc and Ki67 of cells. (ii) Semi-quantification of (i) (*n* = 4 biologically independent samples). Data are represented as mean ± SD. Exact *P* values were calculated by two-tailed Student’s *t*-test and given in the Source Data file. *** *P* < 0.001, ** *P* < 0.01.

**
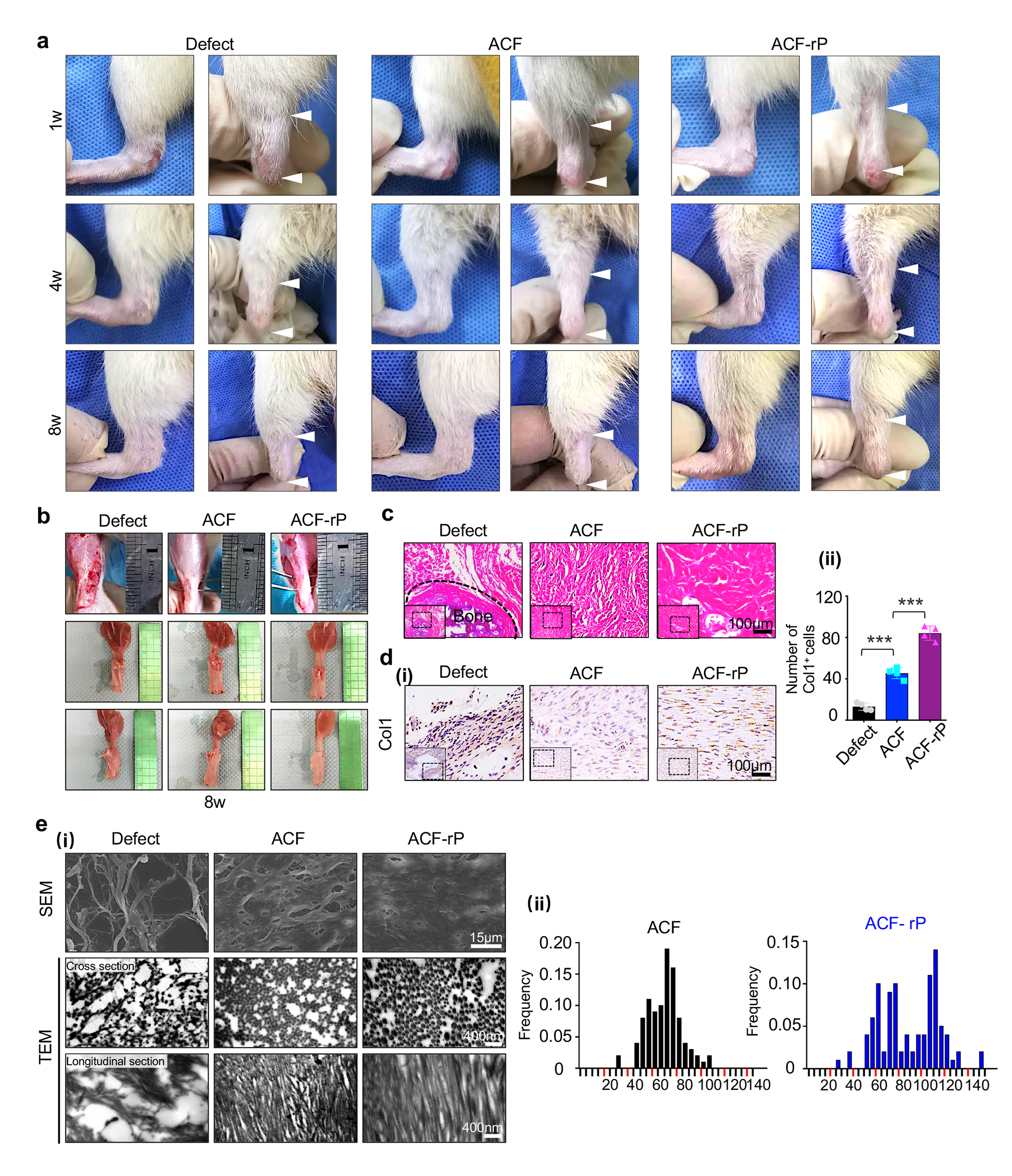
**

**Supplementary Figure 5 Macro-, micro- and nano- structures of neotendons regenerated by ACF loaded with rPOSTN. a** Microscopic images of hindlimb from different groups at 1, 4, and 8 weeks postoperatively. **b** Microscopic images of harvested neotendons at 8 weeks postoperatively. **c** HE staining of cross-sections of neotissues at 8 weeks postoperatively. **d** (i) Immunohistochemistry staining of Col1 of neotendons at 4 weeks postoperatively. (ii) Semi-quantification of (i) (*n* = 4 rats per group*,* one-way ANOVA with Tukey’s post hoc test: *** *P* < 0.001). **e** (i) SEM and TEM (transverse and longitudinal) of newly formed tendon collagen fibrils of each group at 4 weeks postoperatively. (ii) Distribution of collagen fibril diameters in the ACF and ACF-rP groups (*n =* 5 rats per group)*.* Data are represented as mean ± SD. Exact *P* values were given in the Source Data file.

**
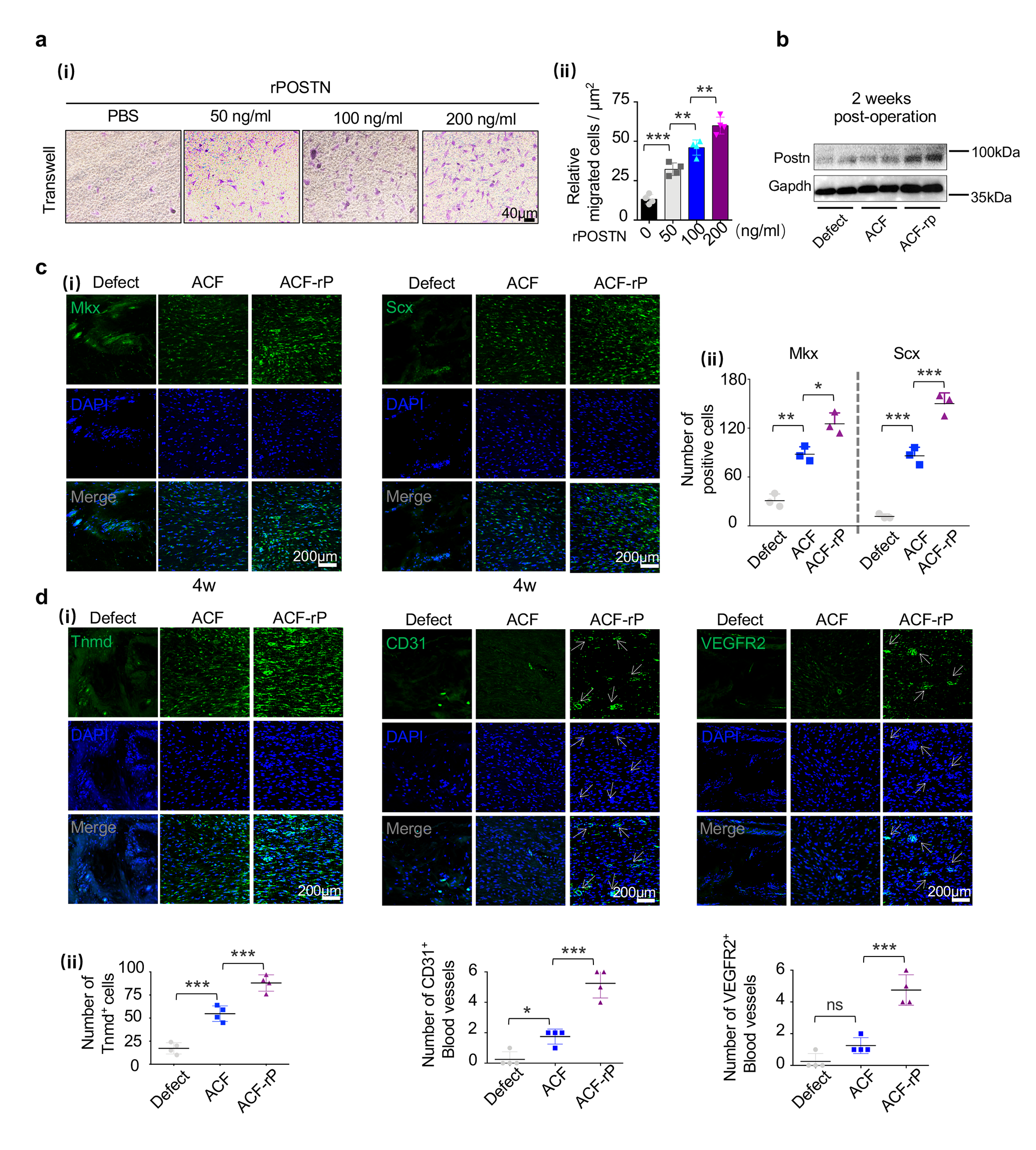
**

**Supplementary Figure 6. rPOSTN facilitates endogenous stem cell recruitment and tenogenesis-specific marker expression. a** (i) Transwell cell migration assay of TSPCs treated by PBS or different concentrations of rPOSTN. (ii) Semi-quantification of (i) (*n* = 4 biologically independent samples). **b** Western blotting of Postn protein levels in neotissues from each group at 2 weeks postoperatively. **c** (i) Immunofluorescence staining of Mkx and Scx of neotendons of each group at 4 weeks postoperatively. (ii) Semi-quantification of (i) (*n =* 3 biologically independent samples). **d** (i) Immunofluorescence staining of Tnmd, CD31, and VEGFR2 of neotendons in each group at 4 weeks postoperatively. (ii) Semi-quantification of (i) (*n =* 4 biologically independent samples). Data are represented as mean ± SD. Exact *P* values were calculated by one-way ANOVA with Tukey’s post hoc test and given in the Source Data file. *** *P* < 0.001, ** *P* < 0.01, * *P* < 0.05, ns: not significant.

.

**
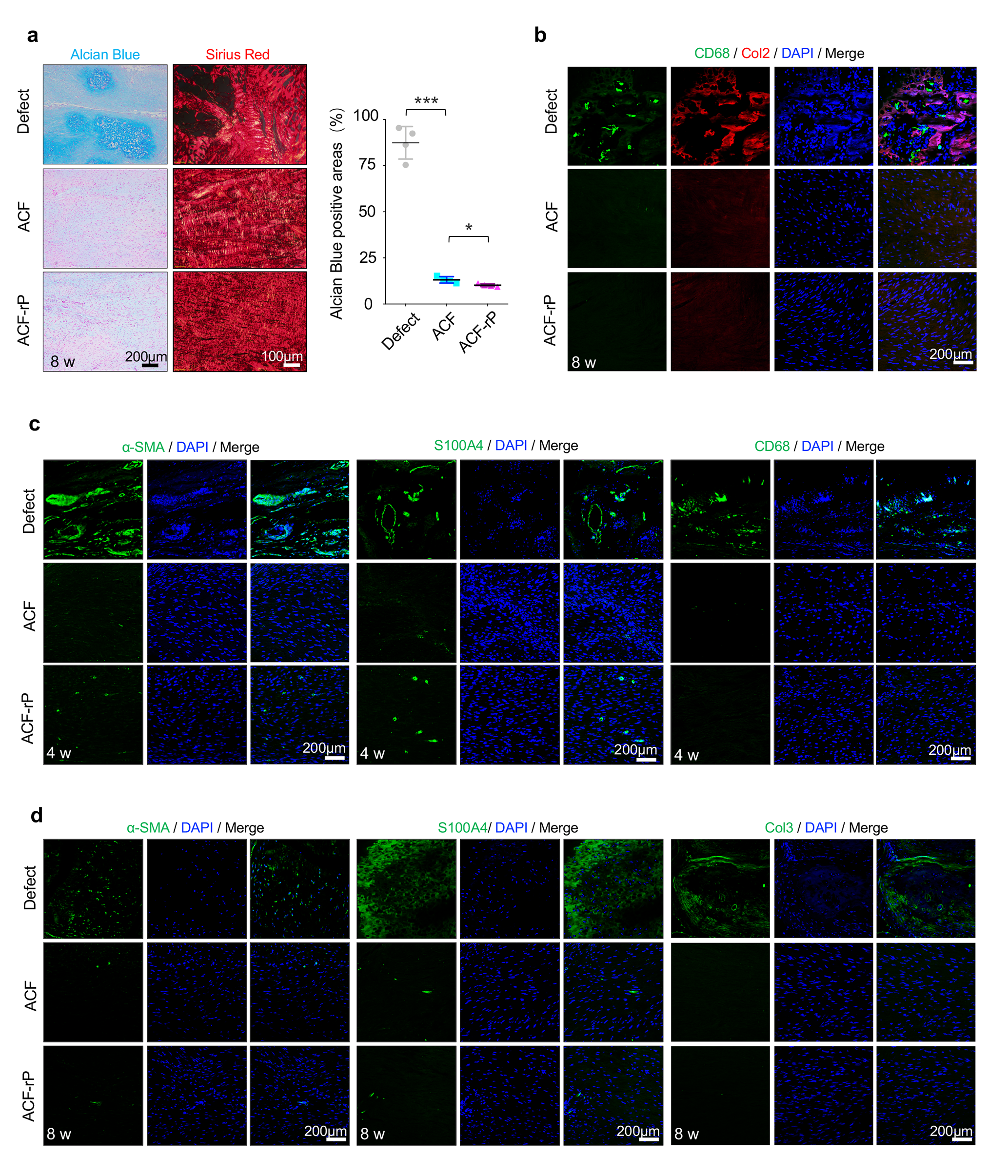
**

**Supplementary Figure 7. No scar or fibrocartilaginous tissues are formed in neotendons from the ACF and ACF-rp groups. a** (i) Alcian Blue and Sirius Red stainings of neotendons of each group at 8 weeks postoperatively. (ii) Semi-quantification of (i) (*n =* 4 rats per group, by two-tailed Student’s *t*-test: *** *P* < 0.001, * *P* < 0.05). **b** Immunofluorescence staining of CD68, Col2 of sections of each group at 8 weeks postoperatively. **c** Immunofluorescence staining of α-SMA, S100A4, CD68 of sections of each group at 4 weeks postoperatively. **d** Immunofluorescence staining of α-SMA, S100A4, Col3 of sections of each group at 8 weeks postoperatively. Data are represented as mean ± SD. Exact *P* values were given in the Source Data file.


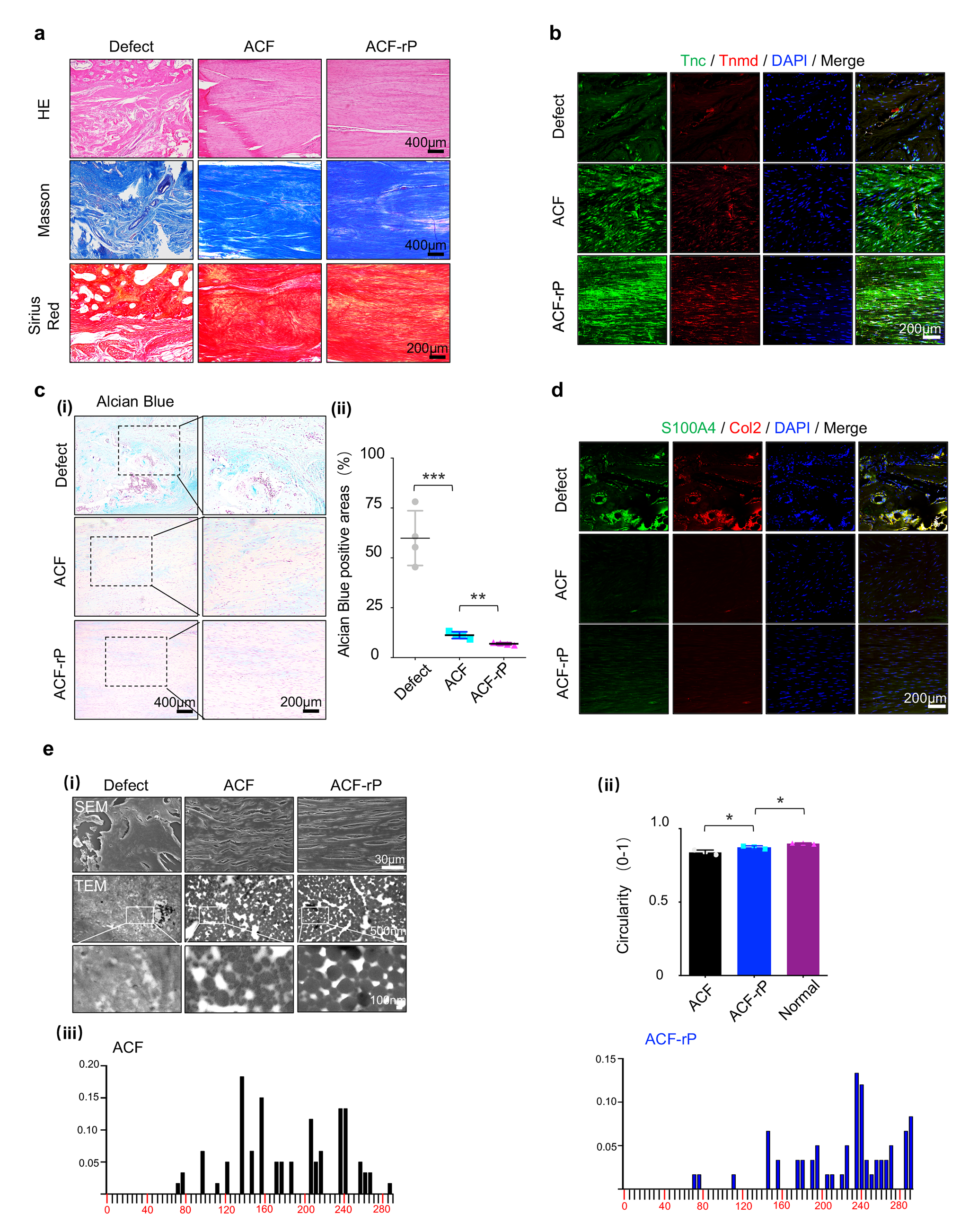


**Supplementary Figure 8. Macro-, micro- and nano- structures of neotendons regenerated by ACF loaded with rPOSTN at 12 weeks postoperatively. a** HE, Masson’s trichrome and Sirius Red stainings of neotendons of each group at 12 weeks postoperatively (*n =* 5 rats per group)*.* **b** Immunofluorescence staining of Tnc, Tnmd of sections of each group at 12 weeks postoperatively. **c** (i) Alcian Blue staining of neotendons of each group at 12 weeks postoperatively. (ii) Semi-quantification of (i) (*n =* 4 rats per group)*.* **d** Immunofluorescence staining of S100A4, Col2 of sections of each group at 12 weeks postoperatively. **e** (i) SEM (longitudinal) and TEM (transverse) of newly formed tendon collagen fibrils of each group at 12 weeks postoperatively. (ii) Collagen circularity of the ACF, ACF-rP and Normal groups. (iii) Distribution of collagen fibril diameters of in the ACF and ACF-rP groups (*n =* 5 rats per group). Data are represented as mean ± SD. Exact *P* values were calculated two-tailed Student’s *t*-test and given in the Source Data file. *** *P* < 0.001, ** *P* < 0.01, * *P* < 0.05.


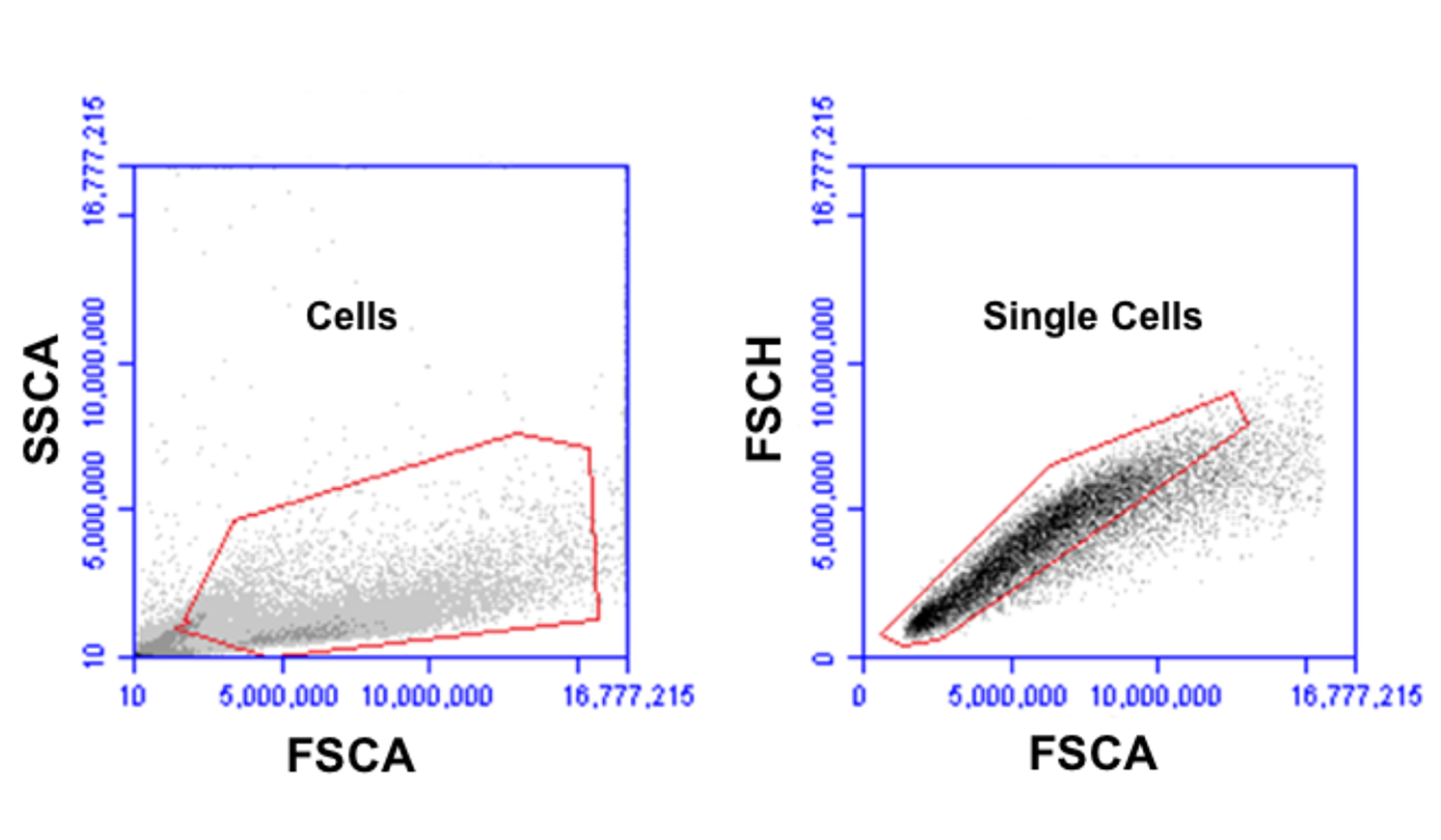
**Supplementary Figure 9.** **Flow cytometry analysis strategy**. Cells were first gated on forward scatter (FSCA) *vs* side scatter (SSCA) to discard cell debris and dead or dying cells. Next FSCH (height) *vs* FSCA (Area) was used to select single cells. CD90, CD105, CD44, CD34 and CD45 antibodies were used for surface staining.

**
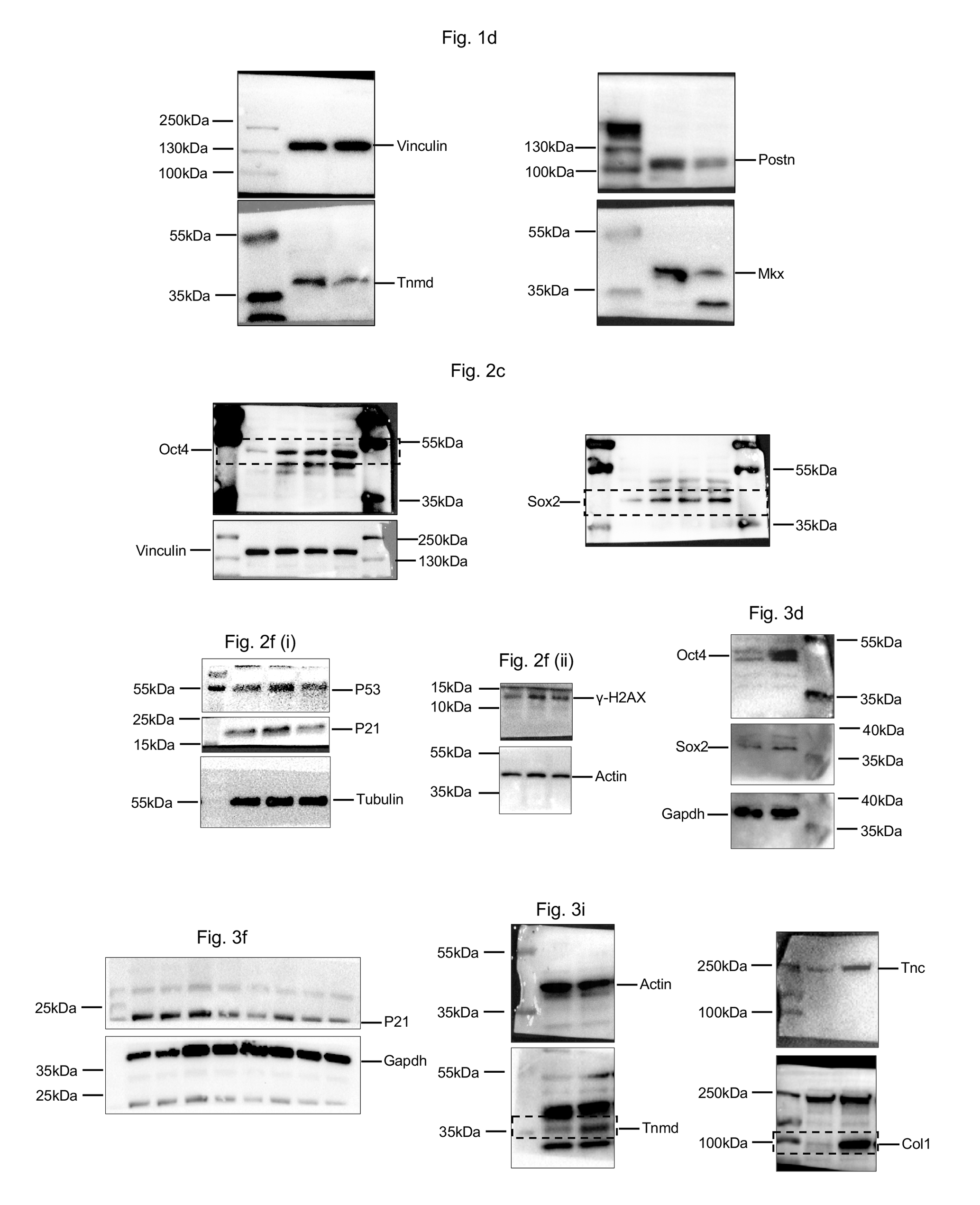
Supplementary Figure 10.** Original scans of the blots in Fig. 1d, 2c, 2f, 3d, 3f and 3i.

**
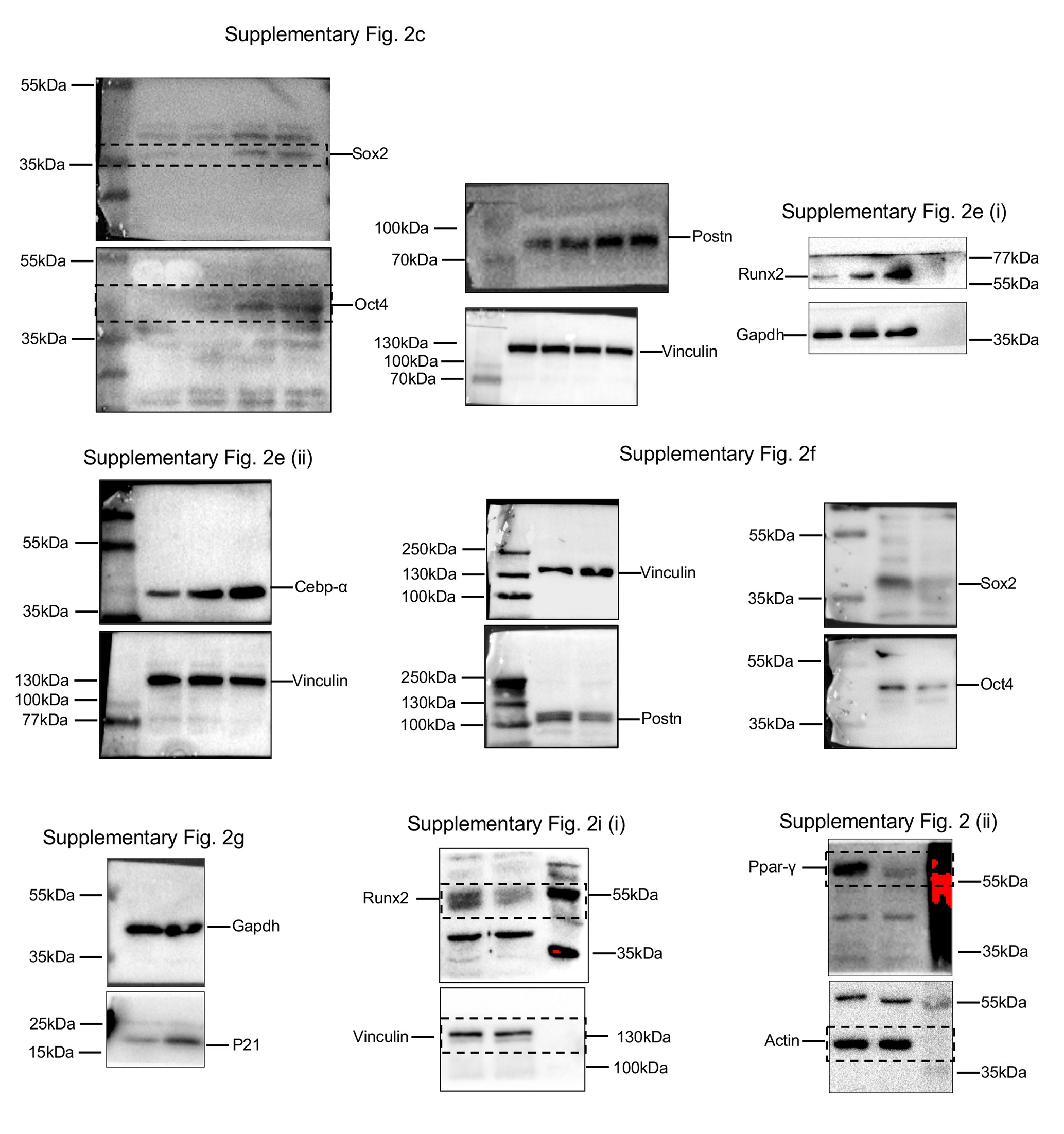
**

**Supplementary Figure 11.** Original scans of the blots in Supplementary Fig. 2c, 2e, 2f, 2g and 2i.

**
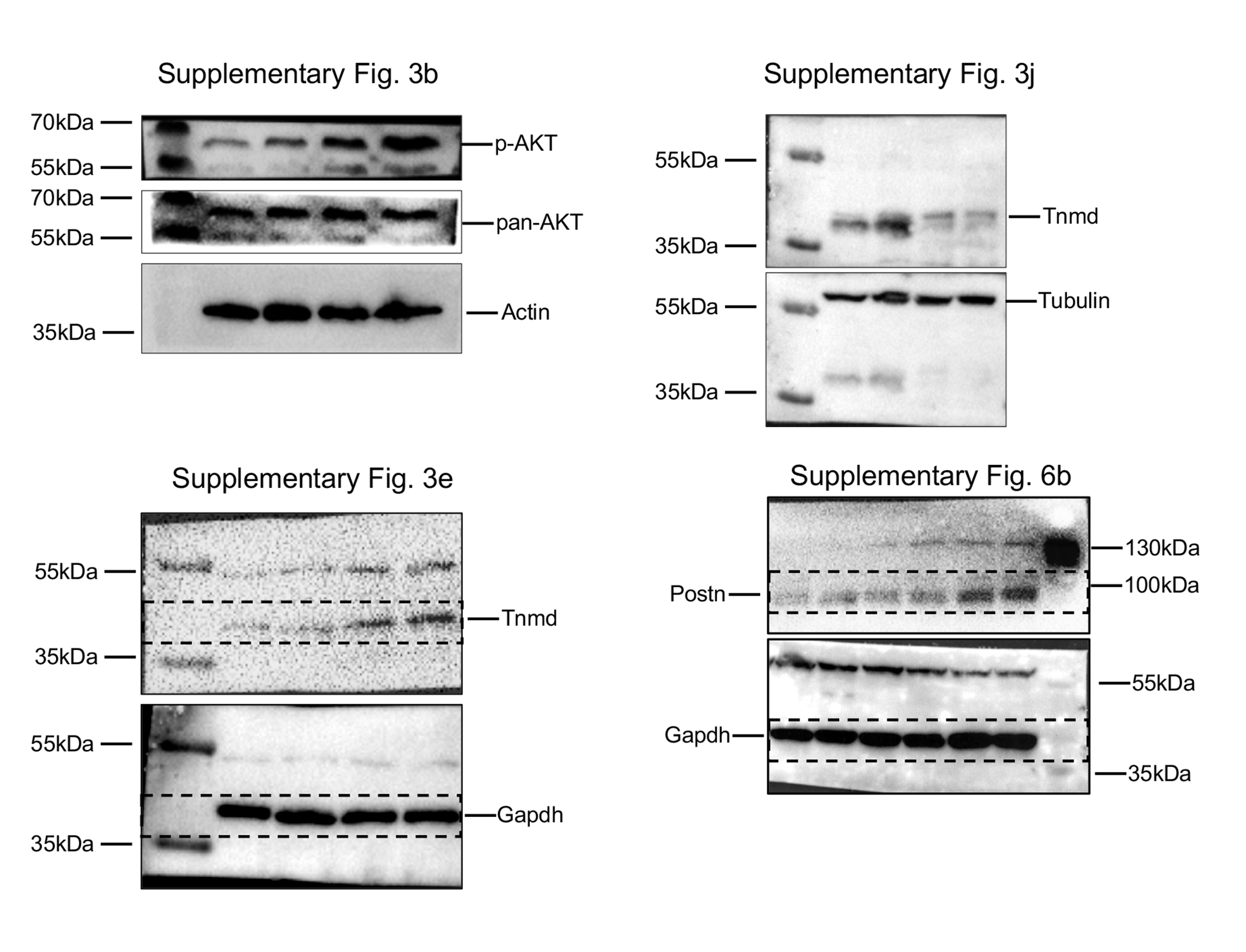
**

**Supplementary Figure 12.** Original scans of the blots in Supplementary Fig. 3b, 3e, 3j and 6b.

**Supplementary Table 1.** Mechanical parameters of ACF.

|  | | Failure force (N) | Failure stress (N/mm^2^) | | Elastic modulus  (N/mm^2^) | Strain at failure (%) | Energy to failure (J/mm^3^) |
| --- | --- | --- | --- | --- | --- | --- | --- |
| ACF | 33.31 ± 2.06 | | | 10.60 ± 0.65 | 11.15 ± 1.08 | 34.54 ± 2.96 | 0.18 ± 0.01 |

*n* ≥ 3 for tensile mechanical testing.

**Supplementary Table 2.** Mechanical parameters of neotissues at 8 weeks postoperatively.

|  | Defect | ACF | ACF-rp | Normal |
| --- | --- | --- | --- | --- |
| Tendon length (mm) | 8.80 ± 0.62 | 11.34 ± 1.05 | 10.00 ± 0.19 | 9.98 ± 0.55 |
| Cross sectional area (mm^2^) | 15.51± 2.73 | 8.15 ± 1.07 | 4.82 ± 0.51 | 4.00 ± 0.52 |
| Failure force (N) | 27.22 ± 7.56 | 44.32 ± 4.32 | 59.55 ± 8.09 | 91.81± 22.77 |
| Stress at failure (MPa) | 1.81 ± 0.61 | 5.77 ± 1.09 | 12.50 ± 2.27 | 22.83 ± 3.34 |
| Modulus (MPa) | 9.62 ± 2.37 | 31.31 ± 5.08 | 51.71 ± 8.01 | 85.05 ± 6.03 |
| Stiffness (N/mm) | 16.65 ± 3.13 | 22.18 ± 4.37 | 24.59 ± 3.88 | 34.27 ± 6.78 |
| Work to failure (J) | 0.078 ± 0.029 | 0.083 ± 0.010 | 0.114 ± 0.019 | 0.128 ± 0.043 |
| Hysteresis (MPa* mm/mm) |  | 0.0151 ± 0.0012 | 0.0203 ± 0.0030 | 0.0317 ± 0.0030 |
| Stress relaxation (%) |  | 57.89 ± 2.70 | 56.51 ± 3.44 | 70.84 ± 6.69 |

Data were presented as mean ± SD, *n* ≥ 3 for tensile mechanical testing.**Supplementary Table 3.** List of primary and secondary antibodies used in the study.

| **REAGENT OR RESOURCE** | **SOURCE** | **IDENTIFIER** | **DILUTION** |
| --- | --- | --- | --- |
| **Antibodies** |  |  |  |
| Rabbit-polyclonal anti-Periostin | Abcam | AB215199 | IF of tissue (1:100) |
| Rabbit-polyclonal anti-Periostin | Abcam | AB92460 | WB (1:1000), IF of tissue (1:100) |
| Rabbit-polyclonal anti-Sox2 | Abcam | AB97959 | WB (1:1000), IF of tissue (1:100), IF of cell (1:200) |
| Rabbit-polyclonal anti-Col2 | Proteintech | 28459-1-AP | IF of tissue (1:100) |
| Rabbit-polyclonal anti-Col3 | Proteintech | 22734-1-AP | IF of tissue (1:100) |
| Rabbit-monoclonal anti-S100A4 | Abcam | AB197896 | IF of tissue (1:200) |
| Rabbit-monoclonal anti-Alpha smooth muscle actin | Abcam | AB124964 | IF of tissue (1:200) |
| Rabbit- monoclonal anti-Phospho-Akt（Ser473） | Cell Signaling  Technology | #4060 | WB (1:1000), IF of cell (1:300) |
| Rabbit-monoclonal anti-Akt（pan） | Cell Signaling  Technology | #4685 | WB (1:1000) |
| Mouse-monoclonal anti-Sox2 | Abcam | AB79351 | IF of tissue (1:200) |
| Goat-polyclonal anti-Oct4 | Abcam | AB27985 | WB (1:1000), IF of tissue (1:100), IF of cell (1:300) |
| Rabbit-polyclonal anti-Oct4 | Abcam | AB181557 | IF of tissue (1:100), IF of cell (1:200) |
| Rabbit-monoclonal anti-Ki67 | Abcam | AB16667 | IHC of tissue (1:200), IF of tissue (1:200),  IF of cell (1:400) |
| Rat-monoclonal anti-Ki67 | Ebioscience | 14-5698-82 | IF of tissue (1:200), IF of cell (1:400) |
| Rabbit-polyclonal anti-P53 | Proteintech | 10442-1-AP | WB (1:1000) |
| Rabbit-monoclonal anti-P21 | Abcam | AB109199 | WB (1:1000) |
| Rabbit-polyclonal anti-γH2AX | Abcam | AB124781 | WB (1:1000), IF of cell (1:400) |
| Rabbit-polyclonal anti-Col1 | Proteintech | 14695-1-AP | WB (1:1000), IF of cell (1:300), IF of tissue (1:100), IHC of tissue (1:100) |
| Rabbit-monoclonal anti-Tenascin-C | Abcam | AB108930 | WB (1:1000), IF of cell (1:300), IF of tissue (1:100) |
| Mouse-monoclonal anti-Tenascin-C | Abcam | AB233198 | WB (1:1000), IF of cell (1:300), IF of tissue (1:100) |
| Rabbit-monoclonal anti-Cebp-α | Abcam | AB40764 | WB (1:1000) |
| Rabbit-polyclonal anti-Temodulin | Abcam | AB203676 | WB (1:1000), IF of cell (1:300), IF of tissue (1:100) |
| Rabbit-polyclonal anti-Mkx | Abcam | AB66939 | WB (1:1000) |
| Mouse-monoclonal anti-Mkx | Santa cruz | Sc-515878 | IF of cell (1:300), IF of tissue (1:100),  IHC of tissue (1:100) |
| Rabbit-polyclonal anti-ScxA | Abcam | AB58655 | IF of tissue (1:100) |
| Mouse-monoclonal anti-Scx | Santa cruz | Sc-518082 | IF of tissue (1:100), IHC of tissue (1:100) |
| Mouse-monoclonal anti-Runx2 | Abcam | AB76956 | WB (1:1000) |
| Rabbit-polyclonal anti-Ppar-γ | Abcam | AB209350 | WB (1:1000) |
| Mouse anti-Actin | ZSGB-BIO | TA-09 | WB (1:3000) |
| Mouse-monoclonal anti-Vinculin | Proteintech | 66305-1-lg | WB (1:3000) |
| Rabbit-polyclonal anti-Beta Tubulin | Proteintech | 10068-1-AP | WB (1:3000) |
| Rabbit-monoclonal anti-Gapdh | Cell Signaling  Technology | #5174 | WB (1:3000) |
| Phalloidin-Fitc (F-actin) | Solarbio | CA1620 | IF of cell (1:500) |
| Rabbit-monoclonal anti-CD34 | Abcam | AB81289 | Flow cyt of cell (1:50) |
| Rabbit-polyclonal anti-CD45 | Abcam | AB10558 | Flow cyt of cell (1:50) |
| Mouse-monoclonal anti-CD105 | Abcam | AB156756 | Flow cyt of cell (1:50) |
| Mouse-monoclonal anti-CD90 | Abcam | AB225 | Flow cyt of cell (1:50) |
| Mouse-monoclonal anti-CD68 | Abcam | AB201340 | IF of tissue (1:100) |
| Rabbit-monoclonal anti-CD44 | Abcam | AB189524 | Flow cyt of cell (1:50) |
| Rabbit-monoclonal anti-CD146 | Abcam | AB75769 | IF of cell (1:300), IF of tissue (1:100) |
| Mouse-monoclonal anti-CD146 | Novus biologicals | SPM620 | IF of tissue (1:100) |
| HRP-linked anti-mouse IgG | Cell Signaling  Technology | 7076S | WB (1:5000) |
| HRP-linked anti-rabbit IgG | Cell Signaling  Technology | 7074S | WB (1:5000) |
| HRP-linked anti-goat IgG | ZSGB-BIO | ZB-2306 | WB (1:5000) |
| FITC-labeled goat  anti-mouse IgG(H + L) | ZSGB-BIO | ZF-0312 | IF (1:300) |
| FITC-labeled goat  anti-rabbit IgG (H + L) | ZSGB-BIO | ZF-0311 | IF (1:300) |
| Rhodamine labeled goat anti-mouse IgG (H + L) | ZSGB-BIO | ZF-0313 | IF (1:300) |
| Rhodamine labeled goat  anti-rabbit IgG (H + L) | ZSGB-BIO | ZF-0316 | IF (1:300) |

IF: immunofluorescence; WB: Western blotting; IHC: immunohistochemistry; Flow cyt: flow cytometry.

**Supplementary Table 4.**  List of reagents or resources used in the study.

| **REAGENT OR RESOURCE** | **SOURCE** | **IDENTIFIER** |
| --- | --- | --- |
| Chemicals and Recombinant Proteins |  |  |
| Recombinant rat Periostin | R&D system | 8994-F2-050 |
| Recombinant human TGFβ1 | Peprotech | AF-100-21C |
| Recombinant mouse GDF-5 | R&D system | 853-G5 |
| Hydrogen peroxide | Sigma-Aldrich | 7722-84-1 |
| LY294002 | Selleck | S1105 |
| Matrigel | Corning | 354234 |
| Senescenceβ-Galactosidase  Staining Kit | Cell Signaling  Technology | #9860 |
| Alcian Blue Stain Kit | Solarbio | G1563 |
| Picro Sirius Red Stain Kit | Abcam | AB150681 |
| Masson’s Trichrome Stain Kit | Solarbio | G1340 |
| Alizarin Red S solution | Solarbio | G1450 |
| Oil Red O Stain Kit | Solarbio | G1262 |
| Crystal Violet Stain solution | Solarbio | G1062 |
| Collagen I, Rat Tail | Corning | 354236 |
| bFGF2 | Peprotech | P13109 |
| EGF | Peprotech | P07522 |
| Mounting Medium with DAPI | ZSGB-BIO | ZLI-9557 |
| Dexamethasone | Sigma-Aldrich | D8893 |
| Dialysis bag | Solarbio | YA1077 |
| DMEM | Hyclone | SH30021.01B |
| Fetal bovine serum (FBS) | Thermo Fisher Scientific | 10099-141 |
| Lipofectamine RNAiMAX  Transfection Reagent | Thermo Fisher Scientific | 13778075 |
| Indomethacin | Sigma-Aldrich | I7378 |
| SYBR Green Supermix | Thermo Fisher Scientific | 4385612 |
| ReverTra Ace qPCR RT Kit | TOYOBO | FSQ-101 |
| TRIzol Reagent | Thermo Fisher Scientific | 15596026 |
| Trypsin-EDTA | Hyclone | SH30042.01 |
| b-Glycerophosphate | APEXBIO | 13408-09-8 |
| L-Ascorbic acid | Sigma-Aldrich | A5960 |
| Penicillin/streptomycin | Thermo Fisher Scientific | 15070063 |
| Collagenase Type I | Thermo Fisher Scientific | 17100017 |
| Dispase | Roche | 10269638001 |
| RIPA Buffer | Thermo Fisher Scientific | 89900 |
| L-Glutamine (200mM) | Thermo Fisher Scientific | 25030081 |

**Supplementary Table 5.** List of primers used in the study.

| TARGET | FOR QPCR DETECTION | |
| --- | --- | --- |
| *Periostin* | FORWARD | TATAAGAGCCCCATTGACTTCGG |
|  | REVERSE | ATTTCCTTCAGTTCCTACCCCAC |
| *Scx* | FORWARD | CTCAGCAACCAGAGAAAGTTGAG |
|  | REVERSE | CTTCACTAGTGGCATCACCTCTT |
| *Mkx* | FORWARD | CTGTCATAGCCAGGTTACCTTGT |
|  | REVERSE | GAGGACAGAGACTTAGCCAGATG |
| *Gapdh* | FORWARD | GGGATATGCAAACCTGGGTTTAG |
|  | REVERSE | GTTATGGGGTCTGGGATGGAATT |

**Supplementary Table 6.** Software and Algorithms.

| Software and Algorithms |  |  |
| --- | --- | --- |
| Nanoscope analysis 1.9 |  | https://www.bruker.com/ |
| Image J (v1.52a) |  |  |
| Horos (version 3, LGPL-3.0) |  | https://www.horosproject.org/ |
| Graph Pad Prism 8.00 |  | https://www.graphpad.com/ |
| Micro-CT Evaluation CTAnsoftware (version 1.15) |  | <http://www.blue-scientific.com/bruker-micro-ct-software/> |
| Instron 5969 50KN |  |  |
| KEGG |  | <https://www.kegg.jp/> |
| GSEA 3.0 |  | http://software.broadinstitute.org/gsea/index.jsp |
| Venny 2.1 |  | http://bioinfogp.cnb.csic.es/tools/venny/ |
| Gene ontology |  | https://geneontology.org/ |
| EPSON Scanner v19. |  |  |
| BD Accuri C6 |  |  |
| Transcriptome Analysis Console (TAC 4.0) |  |  |
