# Supplementary material for: Functional regeneration and repair of tendons using biomimetic scaffolds loaded with recombinant periostin
Source: Nat Commun. 2021 Feb 26;12:1293. doi: 10.1038/s41467-021-21545-1 (PMC7910464; doi:10.1038/s41467-021-21545-1)
Supplement: Supplementary file 2 — Description of Additional Supplementary Files [file 41467_2021_21545_MOESM2_ESM.pdf]

Description of Additional Supplementary Files

Title: Supplementary Video 1

Description: Videos of rat movement in the Defect and ACP-rP groups.
