# Supplementary material for: Functional regeneration and repair of tendons using biomimetic scaffolds loaded with recombinant periostin
Source: Nat Commun. 2021 Feb 26;12:1293. doi: 10.1038/s41467-021-21545-1 (PMC7910464; doi:10.1038/s41467-021-21545-1)
Supplement: Supplementary file 4 — Reporting Summary [file 41467_2021_21545_MOESM4_ESM.pdf]

nature research

Corresponding author(s): Prof. Yan Liu

Last updated by author(s): Jan 18, 2021

Reporting Summary

Nature Research wishes to improve the reproducibility of the work that we publish. This form provides structure for consistency and transparency in reporting. For further information on Nature Research policies, see our Editorial Policies and the Editorial Policy Checklist.

Statistics

For all statistical analyses, confirm that the following items are present in the figure legend, table legend, main text, or Methods section.

n/a	Confirmed	
☐	☒	The exact sample size (n) for each experimental group/condition, given as a discrete number and unit of measurement
☐	☒	A statement on whether measurements were taken from distinct samples or whether the same sample was measured repeatedly
☐	☒	The statistical test(s) used AND whether they are one- or two-sided<br>*Only common tests should be described solely by name; describe more complex techniques in the Methods section.*
☐	☒	A description of all covariates tested
☐	☒	A description of any assumptions or corrections, such as tests of normality and adjustment for multiple comparisons
☐	☒	A full description of the statistical parameters including central tendency (e.g. means) or other basic estimates (e.g. regression coefficient) AND variation (e.g. standard deviation) or associated estimates of uncertainty (e.g. confidence intervals)
☐	☒	For null hypothesis testing, the test statistic (e.g. F, t, r) with confidence intervals, effect sizes, degrees of freedom and P value noted<br>*Give P values as exact values whenever suitable.*
☒	☐	For Bayesian analysis, information on the choice of priors and Markov chain Monte Carlo settings
☒	☐	For hierarchical and complex designs, identification of the appropriate level for tests and full reporting of outcomes
☒	☐	Estimates of effect sizes (e.g. Cohen's d, Pearson's r), indicating how they were calculated

Our web collection on statistics for biologists contains articles on many of the points above.

Software and code

Policy information about availability of computer code

Data collection	Transcriptome Analysis Console (TAC 4.0) ,Image J (v1.52a), KEGG, GSEA(v.3.0), BD Accuri C6, Flowjo(Version 10.5.3), Nanoscope analysis (v.1.9), Instron (5659,50KN),Venny 2.1, EPSON Scanner v19, micro-CT Evaluation CTAn software (version 1.15), Horos (version 3,LGPL-3.0), LSM 5 Release 4.2 software. All softwares and equipments were available in the Methods section and Supplementary Table 6.
Data analysis	Software: Graphpad Prism; version 8.00. Presentation of all statistical analyses was available in Methods section, chapter"Statistical analysis".

For manuscripts utilizing custom algorithms or software that are central to the research but not yet described in published literature, software must be made available to editors and reviewers. We strongly encourage code deposition in a community repository (e.g. GitHub). See the Nature Research guidelines for submitting code & software for further information.

Data

Policy information about availability of data

All manuscripts must include a data availability statement. This statement should provide the following information, where applicable:

- Accession codes, unique identifiers, or web links for publicly available datasets
- A list of figures that have associated raw data
- A description of any restrictions on data availability

The RNA-seq datasets have been submitted to the NCBI database under the accession number GSE158342.Other databases used in the study are GENE ONTOLOGY online database (http://geneontology.org/) and GSEA online database (https://www.gsea-msigdb.org/gsea/index.jsp). The authors declare that all other data supporting the findings of this study are avaliable within the article and its Supplementary information files. The source data are provided with this paper.

Field-specific reporting

Please select the one below that is the best fit for your research. If you are not sure, read the appropriate sections before making your selection.

☒ Life sciences ☐ Behavioural & social sciences ☐ Ecological, evolutionary & environmental sciences

For a reference copy of the document with all sections, see nature.com/documents/nr-reporting-summary-flat.pdf

Life sciences study design

All studies must disclose on these points even when the disclosure is negative.

Sample size	No statistical method was used to predetermine the sample size. Sample size was determined according to previously published papers in related field and prior experience in our laboratory. Rat and mice sample size was estimated by pilot experiments that presented trends of effects and their sizes. In most cases, an n=5 was the minimal amount used, only where effect size was large an n=3 yielded significant results. Specific details were available in Methods section, chapter "In vivo subcutaneously transplantation", "Tendon injury and repair animal model".
Data exclusions	The only data excluded were those experiments with microbial contamination for primary rat TSPCs (Tendon stem/progenitor cells) isolation and culture. Because animals are gated, rats and mice were excluded for poor body condition (e.g., weight loss, serious injuries) including the subcutaneous transplantation of scaffold material loaded with TSPCs in BALB/c mice and scaffold material transplantation in Achilles tendon defect in SD rats. Exclusion criteria were pre- established before the study. No sample or animals were excluded from the analysis.
Replication	At least three biological independent samples were performed for each experiment. All experiments were performed as technical or biological replications as appropriate for the experimental design.
Randomization	All animal were randomly assigned to experimental or control groups.
Blinding	All the surgical treatments and the functional experiments were done by blinded investigators. Blinding was also performed later in the outcomes collection and assessment by other investigators.

Reporting for specific materials, systems and methods

We require information from authors about some types of materials, experimental systems and methods used in many studies. Here, indicate whether each material, system or method listed is relevant to your study. If you are not sure if a list item applies to your research, read the appropriate section before selecting a response.

Materials & experimental systems

n/a	Involved in the study	
☐	☒	Antibodies
☐	☒	Eukaryotic cell lines
☒	☐	Palaeontology and archaeology
☐	☒	Animals and other organisms
☒	☐	Human research participants
☒	☐	Clinical data
☒	☐	Dual use research of concern

Methods

n/a	Involved in the study	
☒	☐	ChIP-seq
☐	☒	Flow cytometry
☒	☐	MRI-based neuroimaging

Antibodies

Antibodies used	All antibodies were listed and in detail described in Supplementary Table 3 and See Methods, chapter "Western blotting" and "Immunohischemistry and Immunofluorescence".Detailed dilutions of all antibodies are described in Supplementary Table 4.
Validation	Rabbit-polyclonal anti-Periostin (#ab215199, Abcam): Application: IHC-Fr, IHC-P Species Reactivity: Mouse, Rat, Human<br>Rabbit-polyclonal anti-Periostin (#ab92460, Abcam): Application: WB ;Species Reactivity: Mouse, Rat, Human<br>Rabbit-polyclonal anti-Sox2 #ab97959, Abcam) : Application: ICC, IHC-P, WB; Species Reactivity: Mouse, Human (predicted: Rat, Sheep, Horse, Chicken, Cow, Pig)<br>Rabbit-polyclonal anti-Col2 (#28459-1-AP, Abcam) :Application: WB, IHC, IF, ELISA ; Species Reactivity: Human, mouse, rabbit, rat<br>Rabbit-polyclonal anti-Col3(#22734-1-AP, Proteintech) : Application: WB, IP, IHC, IF, ELISA ;Species Reactivity: human, mouse, rat, canine<br>Rabbit-monoclonal anti-S100A4 (#197896, Abcam): Application: Flow Cyt, ICC/IF, IHC-P, IP, WB ; Species Reactivity: Mouse, Rat, Human<br>Rabbit-monoclonal anti-Alpha smooth muscle actin (#124964, Abcam): Application: Flow Cyt, ICC/IF, IHC-P, WB ;Species Reactivity: Mouse, Rat, Human<br>Rabbit- monoclonal anti-Phospho-Akt(Ser473)(#4060, CST): Application: WB,IP, IHC-P. IF, Flow cyt ;Species Reactivity: Mouse, Rat, Human

Rabbit- monoclonal anti-Akt pan) (#4685,CST): Application: WB, IP, IHC-P. IF, Flow cyt ; Species Reactivity: Mouse, Rat, Human
Mouse-monoclonal anti-Sox2(#79351 Abcam): Application: Flow Cyt, ICC/IF, WB ; Species Reactivity: Mouse, Human, Apteronotus leptorhynchus (predicted: Rat, Sheep, Horse, Chicken, Cow, Pig)
Goat-polyclonal anti-Oct4 (#27985, Abcam): Application: WB; Species Reactivity: Mouse, Rat, Human
Rabbit-polyclonal anti-Oct4 #181557 Abcam) :Application: ChIP, Flow Cyt, ICC/IF, IHC-P, IP, WB ;Species Reactivity: Mouse, Human (predicted: Rat, Sheep, Horse, Cow, Pig
Rabbit-monoclonal anti-Ki67 (#16667, Abcam): Application: Flow Cyt, ICC, IHC-P, IHC, WB ; Species Reactivity: Mouse, Rat, Human
Rat-monoclonal anti-Ki67 #14-5698-82 Ebioscience) :Application: Flow Cyt, ICC/IF, IHC-P, WB ; Species Reactivity: Mouse, Rat, Human
Rabbit-polyclonal anti-P53 (#10442-1-AP, Proteintech): Application: WB, IP, IF, CoIP, chIP, ELISA; Species Reactivity: human, rat, mouse, Artemia, goat, sheep, swine
Rabbit-monoclonal anti-P21(#109199,Abcam): Application: WB ; Species Reactivity: Rat, Human
Rabbit-polyclonal anti-γ-H2AX #124781,Abcam) : Application: Flow Cyt, ICC/IF, IHC-P, IP, WB ;Species Reactivity: Mouse, Rat, Human
Rabbit-polyclonal anti-Col1(#14695-1-AP, Proteintech): Application: WB, IHC, IF,ELISA ;Species Reactivity: mouse, human, pig, rat, canine, rabbit, swine, toad
Rabbit-monoclonal anti-Tenascin-C (#108930,Abcam): Application: IHC-Fr, IHC-P, WB ; Species Reactivity: Mouse, Rat, Human
Mouse-monoclonal anti-Tenascin-C (#ab233198,Abcam): Application: IHC-P, WB; Species Reactivity: Rat (predicted: Mouse, Human)
Rabbit-monoclonal anti-Cebp-α (#40764 Abcam) : Application: WB; Species Reactivity: Mouse, Rat, Human
Rabbit-polyclonal anti-Temodulin (#203676,Abcam): Application: IHC-P, WB; Species Reactivity: Mouse, Rat, Human
Rabbit-polyclonal anti-Mkx (#66939,Abcam): Application: Species WB; Reactivity: Mouse (predicted: Mouse, Rabbit)
Mouse-monoclonal anti-Mkx (#sc-515878, Santa Cruz): Application: IF, IP, WB ;Species Reactivity: Rat, Human,Mouse
Rabbit-polyclonal anti-ScxA(#58655,Abcam): Application: WB ;Species Reactivity:Mouse
Mouse-monoclonal anti-Scx (#sc-518082, Santa Cruz): Application: IF, IHC-P, WB ;Species Reactivity: Rat, Human,Mouse
Mouse-monoclonal anti-Runx2 (#76956,Abcam): Application: Flow Cyt, ICC/IF, WB; Species Reactivity: Mouse, Rat, Human
Rabbit-polyclonal anti-Ppar-γ (#209350,Abcam) : Application: ICC/IF, WB; Species Reactivity: Mouse, Rat, Human, Pig
Mouse anti-ACTIN (#TA-09, ZSGB-BIO) : Application: WB ; Species Reactivity: Human, mouse, rat, monkey, dog
Mouse-monoclonal anti-Vinculin(#66305-1-lg, Proteintech): Application: WB, IHC, IF, FC, CoIP, ELISA ; Species Reactivity: human, mouse, rat, pig, hamster
Rabbit-polyclonal anti-Beta Tubulin(#10068-1-AP, Proteintech): Application: WB, IP, IHC, IF, ELISA; Species Reactivity: human, mouse, rat
Rabbit-monoclonal anti- GAPDH(#5174, CST): Application: WB,IHC,IF ;Species Reactivity: human, mouse, rat,Monkey
Phalloidin-Fitc F-actin #CA1620, Solarbio) : Application: IF ;Species Reactivity:Human,mouse,rat
Rabbit-monoclonal anti-CD34(#81289 Abcam) : Application: Flow Cyt, ICC/IF, IHC-Fr, IHC-P, IP, WB; Species Reactivity: Mouse, Rat, Human
Rabbit-polyclonal anti-CD45 (#10558 Abcam) : Application: Flow Cyt, IHC-P, WB; Species Reactivity: Mouse, Rat, Human
Mouse-monoclonal anti-CD105 (#156756,Abcam) : Application: Flow Cyt, ICC/IF, WB; Species Reactivity: Mouse, Rat, Dog, Human, Monkey
Mouse-monoclonal anti-CD90 (#225, Abcam): Application: Flow Cyt, ICC, WB ;Species Reactivity: Rat (predicted: Mouse, Rabbit, Horse)
Mouse-monoclonal anti-CD68 (#201340,Abcam) : Application: Flow Cyt, ICC/IF, IHC-P, WB ;Species Reactivity: Mouse, Rat, Human
Rabbit-monoclonal anti-CD44 (#189524, Abcam): Application: Flow Cyt, ICC/IF, IHC-P, IP, WB ;Species Reactivity: Mouse, Rat, Human
Rabbit-monoclonal anti-CD146 (#75769, Abcam): Application: Flow Cyt, ICC/IF, IHC-P, WB ;Species Reactivity: Mouse, Rat, Human
Mouse-monoclonal anti-CD146 (#SPM620, Novus biologicals): Application: Flow Cyt, ICC/IF, IHC-P, WB; Species Reactivity: Rat, Human

Eukaryotic cell lines

Policy information about cell lines

Cell line source(s)	Cells used in our study were isolated from Achilles tendon in postnatal 1 day and adult 6-8 week SD rats. Details were available in Methods section, chapter "Cell isolation and expansion" and "Monoclonal selection".
Authentication	Rat TSPCs were isolated according to well-established protocols. Furthermore, we utilized flow cytometry to demonstrate that the isolated TSPCs expressed mesenchymal surface markers, including CD90, CD105 and CD44, but were negative for hematopoietic cell markers CD45 and CD34. They were able to produce colonies in vitro, possessed osteogenic, adiopogenic and chondrogenic differentiation capacity. At least three biological replicates were preformed for each donor cells to confirm the consistency of the cell lines.
Mycoplasma contamination	All cell lines were negative for mycoplasma.

Commonly misidentified lines (See ICLAC register)	No commonly misidentified cell lines were used in the study.

Animals and other organisms

Policy information about studies involving animals; ARRIVE guidelines recommended for reporting animal research

Laboratory animals	Male Sprague Dawley neonatal (1 day, body weight of 1.5-2 g) and adult rats (6 weeks, body weight of 200-250 g) were obtained from Weitong Lihua Experimental Animal Center (China).<br>6-8-week-old male BALB/c nude mice were obtained from Weitong Lihua Experimental Animal Center (China).<br>Temperature(23 ± 2 °C) and humidity (55%) were held constant in animal housing.
Wild animals	No wild animals were used.
Field-collected samples	No samples collected from the field were used.
Ethics oversight	All experimental procedures used in this study were performed in compliance with animal welfare ethical regulations and approved by the Animal Use and Care Committee of Peking University (LA2018302).

Note that full information on the approval of the study protocol must also be provided in the manuscript.

Flow Cytometry

Plots

Confirm that:

- [x] The axis labels state the marker and fluorochrome used (e.g. CD4-FITC).
- [x] The axis scales are clearly visible. Include numbers along axes only for bottom left plot of group (a 'group' is an analysis of identical markers).
- [x] All plots are contour plots with outliers or pseudocolor plots.
- [x] A numerical value for number of cells or percentage (with statistics) is provided.

Methodology

Sample preparation	The primary TSPCs were isolated from fresh Achilles tendon of 6-8 week SD rats without receiving any special treatment . After primary culture and monoclonal selection,we incubated 1x10^6 TSPCs respectively with primary antibodies of CD90, CD105, CD44, CD45 and CD34 for 1 h and fluorescent secondary antibody for 1 h at 4 °C, then analyzed the samples using flow cytometer (BD BD Accuri C6) to calculate the expression of cell surface markers in isolated TSPCs. More details were available in Methods section.
Instrument	BD Accuri C6 Flow cytometer.
Software	BD Accuri C6 Software.
Cell population abundance	N/A. We did not perform cell sorting for specific cell population. We identified the primary TSPCs using flow cytometry to confirm mesenchymal surface marker markers CD90, CD105 and CD44, and exclude hemopoietic stem cell markers CD45 and CD34.
Gating strategy	Cells were first gated on forward scatter (FSCA) vs side scatter (SSCA) to discard cell debris and dead or dying cells. Next FSCH (height) vs FSCA (Area) was used to select single cells.

- [x] Tick this box to confirm that a figure exemplifying the gating strategy is provided in the Supplementary Information.
